# Mitogenomic Insights into Orthocladiinae (Diptera: Chironomidae): Structural Diversity and Phylogenetic Implications

**DOI:** 10.3390/biology14091178

**Published:** 2025-09-02

**Authors:** Hai-Feng Xu, Xiu-Ru Xiao, Zhi-Chao Zhang, Yu-Fan Li, Xiao-Long Lin

**Affiliations:** 1 Engineering Research Center of Environmental DNA and Ecological Water Health Assessment, Shanghai Ocean University, Shanghai 201306, China; haifengxu2024@gmail.com (H.-F.X.); zyxr1023@hotmail.com (X.-R.X.); zzc514644@gmail.com (Z.-C.Z.); liyufan916@gmail.com (Y.-F.L.); 2 Shanghai Universities Key Laboratory of Marine Animal Taxonomy and Evolution, Shanghai Ocean University, Shanghai 201306, China

**Keywords:** mitogenome, rearrangement, NGS

## Abstract

Mitochondrial genomes are powerful tools for taxonomic delimitation and species identification, yet they remain scarce for Chironomidae (Diptera). In this study, we assembled and annotated 63 new mitochondrial genomes, encompassing 63 species within 39 genera in Orthocladiinae *sensu lato* (including Prodiamesinae and Orthocladiinae) and Chironominae by whole-genome sequencing, marking the first report of mitochondrial genome data for the Xiaomyini. Comparative analyses revealed structural variation, including transfer RNA gene rearrangements, along with strong nucleotide composition bias, codon usage patterns, and gene-specific selection pressure differences. Distinct evolutionary dynamics were detected among protein-coding genes, ribosomal RNAs, transfer RNAs, and the control region. Heterogeneity analyses and phylogenetic analyses showed that amino acid datasets perform better for basal branch of Orthocladiinae relationships, although the resolution within non-basal branches of Orthocladiinae remains limited. By substantially increasing both the number and taxonomic breadth of mitochondrial genomes in Chironomidae, this study delivers a vital foundation for future multi-marker phylogenetic reconstruction, taxonomic revision, and rapid species identification, with direct applications to biodiversity conservation and freshwater ecosystem monitoring.

## Simple Summary

This study assembled and analyzed 63 new mitochondrial genomes from three key chironomid subfamilies (Orthocladiinae, Prodiamesinae and Chironominae), significantly expanding genetic resources for this ecologically important insect family. The research uncovered variations in genome structure, nucleotide composition, and evolutionary pressures across different mitochondrial genes. The amino acid data can support phylogenetic reconstruction for certain taxa, but still has limited analytical power for closely related species differentiation and small-sample groups. By providing a wealth of new genomic data, this work supports future studies on chironomid biodiversity, phylogenetics, and species identification—aiding conservation and freshwater ecosystem monitoring efforts.

## 1. Introduction

The mitochondrial genome (mitogenome), as an important molecular marker, is characterized by maternal inheritance (though recent instances of paternal inheritance have been reported in insects including dipterans), a relatively fast evolutionary rate, and a low recombination rate [1,2,3,4,5,6]. A typical insect mitogenome is a circular double-stranded DNA molecule approximately 14–20 kb in length, with a highly conserved gene composition usually comprising 37 coding units, with 13 protein-coding genes (PCGs), two ribosomal RNA genes (rRNAs), 22 transfer RNA genes (tRNAs), and a non-coding control region (CR) [7]. Although mitogenomes have been widely used in species identification, phylogeographic studies, and shallow-level phylogenetic analyses, they still have limitations in resolving higher-level phylogenetic relationships, such as signal saturation and lack of sufficient conserved sites [8]. Therefore, mitochondrial data are often combined with other data (e.g., nuclear genes or morphological data) to improve the robustness of phylogenetic inferences [9,10]. Nevertheless, as a fundamental molecular dataset, the mitogenome retains the advantages of ease of acquisition and high information density, making it particularly suitable for preliminary taxonomic delimitation and species identification [11,12,13,14,15,16,17].

**Figure 1:**
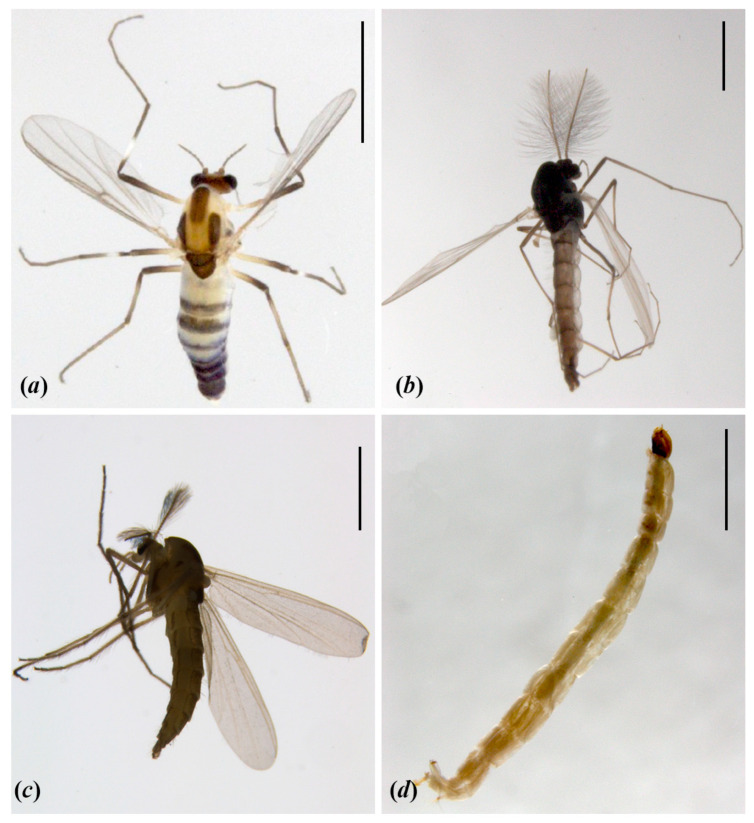
Photos of Orthocladiinae and Prodiamesinae: (**a**) adult female of *Cricotopus flavozonatus*; (**b**) adult male of *Diplocladius cultriger*; (**c**) adult male of *Euryhapsis fuscipropes*; (**d**) larva of *Propsilocerus paradoxus*. Scale bars: 1 mm in (**a**–**c**); 2 mm in (**d**).

The Chironomidae (Diptera) represents one of the most diverse lineages within Diptera, with more than 7800 species described globally (Figure 1) [18,19,20]. Most larvae of this family inhabit a wide range of freshwater environments, where they constitute a dominant component of benthic macroinvertebrate communities. Owing to their ecological sensitivity and wide distribution, chironomids are widely recognized as effective bioindicators for assessing water quality and monitoring freshwater ecosystem health [21]. The Orthocladiinae, one of the most diverse subfamilies within Chironomidae, exhibits an extremely wide distribution, occurring from tropical to polar regions and inhabiting environments ranging from typical freshwater habitats to extreme conditions such as hypersaline lakes and groundwater systems [19,22,23]. However, this subfamily shows pronounced morphological convergence and conservative traits, making it difficult for traditional taxonomy to effectively resolve its phylogenetic relationships, and leading to long-standing controversies in the delimitation of higher-level taxa (e.g., genera and tribes) [24,25,26]. Although molecular systematics has provided new perspectives for Orthocladiinae taxonomy in recent years, most existing studies are based on short-fragment markers (e.g., *COI*, *ITS*), which offer limited resolution [27,28]. While the mitogenome alone cannot fully resolve deep phylogenetic relationships, its complete set of genes and structural variation can provide supplementary evidence for taxonomic delimitation and help identify potential hypervariable regions for subsequent multi-gene analyses [12,29]. However, publicly available mitogenome data for Orthocladiinae remain extremely scarce, severely limiting related research.

In this study, we obtained 52 complete and 11 incomplete mitogenome data (63 species within 39 genera) for multiple representative taxa of Orthocladiinae, Prodiamesinae, and Chironominae by whole-genome sequencing. We focused on analyzing their structural characteristics, nucleotide composition bias, and evolutionary rate differences, and further assessed the utility of mitogenomes in genus-level classification within Orthocladiinae by integrating existing data. This work aimed to fill the current gap in Orthocladiinae mitogenome resources, provide a foundation for future multi-marker integrative phylogenetic reconstruction, and offer reference data for rapid species identification in aquatic ecological monitoring.

## 2. Materials and Methods

### 2.1. Sample Collection and DNA Extraction

From 2011 to 2021, a total of 63 species were collected from the field in China, Italy, and Namibia using Malaise traps, light traps, and D-nets (Table S1). Preservation methods varied according to life stage: adult specimens were fixed in 85% ethanol, whereas larval specimens were preserved in 95% ethanol. All samples were stored at −20 °C in darkness. Taxonomic identification of specimens was conducted at Shanghai Ocean University, China, with reference to the relevant taxonomic literature [24,30,31,32,33,34,35,36]. Morphological examinations were performed using stereomicroscopy, supplemented by compound microscopy for dissected specimens. Male adults were identified morphologically by Professor Lin, while female adults, larvae, and species-level determinations were primarily based on *COI* barcode sequencing and BOLD database matching. All identification results were rigorously verified by our research team through dual validation procedures. Subsequently, the head–thorax exoskeleton was cleansed and mounted in Euparal^®^ on the same microscope slide alongside its corresponding body parts [22].

The abdomens of adult specimens and the thoracic segments of larvae were used for DNA extraction by using two commercial kits: the Qiagen DNA Blood and Tissue Kit (Qiagen, Hilden, Germany) and the Universal Genomic DNA Kit (CWBIO, Taizhou, China). All procedures strictly followed the manufacturers’ protocols. Voucher specimens are deposited in the College of Fisheries and Life Science, Shanghai Ocean University, China.

Based on a foundation of 63 species (each represented by one sequence), we incorporated 41 published mitochondrial genomes for the phylogenetic analysis, resulting in a total of 104 samples. The ingroup Orthocladiinae *sensu lato* (Orthocladiinae + Prodiamesinae) included 45 genera (80 species), of which 42 genera (75 species) belonged to Orthocladiinae and three genera (five species) to Prodiamesinae. Among these, the mitogenomes of 58 species were newly sequenced in this study, while the remaining 22 species’ sequences were sourced from GenBank. The Chironominae was selected as the outgroup for being the closest sister lineage to Orthocladiinae, represented by 24 species from four tribes (Chironomini, Pseudochironomini, Xiaomyini, and Tanytarsini) [28]. Within Chironominae, five species were newly sequenced, and 19 were retrieved from GenBank. Detailed accession number information of all downloaded sequences can be found in Table S2 [37,38,39,40,41,42,43,44,45,46,47,48,49,50].

### 2.2. Sequencing, Assembly, and Annotation

A multi-step approach was employed to ensure the accuracy of the mitochondrial genome sequencing and assembly. First, the *COI* barcode fragment of each sample was amplified and sequenced using the universal primers LCO1490 and HCO2198 [51] following the PCR protocol of the previous study [52]. Amplification products were purified and subjected to both Sanger sequencing and high-throughput sequencing, providing dual validation for species identification and subsequent genome assembly.

Whole-genome sequencing was performed by Novogene Co., Ltd. (Beijing, China) on an Illumina NovaSeq 6000 platform (Illumina, San Diego, CA, USA), generating 150 bp paired-end reads. Raw reads were quality-trimmed using Trimmomatic v0.32 [53], yielding approximately 3 Gb of high-quality data per sample. De novo assembly of the mitogenomes employed two complementary strategies: NovoPlasty v4.3.1 (k-mer = 39, Brussels, Belgium) [54] and IDBA-UD (k-mer range 40–120, Hong Kong, China) [55]. The assembled contigs were screened in Geneious Prime v2024.0.5 (Biomatters, Auckland, New Zealand) via BLAST v2.16.0 using the obtained *COI* sequences as references, and mapping of clean reads was performed to validate the assembly accuracy.

Gene annotation was conducted using the MITOS2 web server [56] (https://usegalaxy.eu, accessed 12 July 2025) on the Galaxy platform, applying the invertebrate mitochondrial genetic code and the RefSeq89 Metazoa database. Annotation files in BED format generated by MITOS2 were manually checked and refined in Geneious Prime. All newly sequenced mitogenomes have been deposited in the GenBank database under accession numbers PX067949–PX06800 and PV994456–PV994466.

### 2.3. Composition Analyses, RSCU, and Evolutionary Rate

Molecular features and evolutionary dynamics of the mitochondrial genomes were characterized through multi-dimensional analyses. Nucleotide composition of the whole genomes and individual functional genes was assessed using SeqKit v2.3.0 (Shenzhen, China) [57]. The base composition skewness was calculated as AT-skew = (A − T)/(A + T) and GC-skew = (G − C)/(G + C) to quantify nucleotide bias. Codon usage patterns of the 13 PCGs were analyzed in MEGA v12 (Philadelphia, PA, USA) [58], and relative synonymous codon usage (RSCU) values were calculated to detect species-specific codon preferences.

Selective pressure was evaluated in DnaSP v6.12.01 (Barcelona, Spain) [59] using the “mtDNA Drosophila” genetic code table, calculating the synonymous substitution rate (Ks), non-synonymous substitution rate (Ka), and their ratio (Ka/Ks) for each PCG. Finally, mitochondrial genome structures were visualized using the Proksee v1.0.1 (Edmonton, AB, Canada) [60] platform to provide intuitive structural comparisons for comparative genomic analyses.

### 2.4. Phylogenetic Analyses

A combined dataset strategy was adopted to comprehensively resolve phylogenetic relationships, and all analyses were based on the sequences of 13 PCGs and two rRNA genes.

Protein sequences of the 13 PCGs and nucleotide sequences of the two rRNA genes were aligned separately using MAFFT v7.526 (Kyoto, Japan) [61]. Alignments were refined with trimAl v1.4.1 (Barcelona, Spain) [62] using the “–automated1” option to remove poorly aligned positions and gaps. The amino acid alignments of PCGs were back-translated into the corresponding nucleotide sequences. Based on these alignments, five complementary datasets were concatenated using FASconCAT-G v1.06.1 (Santa Cruz, CA, USA) [63]: (1) AA—amino acid sequences of the 13 PCGs; (2) PCG123—nucleotide sequences of all codon positions of the 13 PCGs; (3) PCG123_rRNAs—nucleotide sequences of the 13 PCGs and two rRNAs; (4) PCG12—nucleotide sequences of the first and second codon positions of the 13 PCGs; (5) PCG12_rRNAs—nucleotide sequences of the first and second codon positions of the 13 PCGs plus the two rRNAs. Sequence heterogeneity for each dataset was assessed using AliGROOVE v1.08 (Bonn, Germany) [64].

Phylogenetic reconstruction was performed using the maximum likelihood (ML) method in IQ-TREE v2.3.6 (Vienna, Austria) [65]. The best-fit substitution models for each dataset were automatically selected by ModelFinder [66], and node support was evaluated with 1000 bootstrap replicates to ensure the robustness of the phylogenetic inference.

## 3. Results and Discussion

### 3.1. Nucleotide Composition

A comparative analysis of 63 mitochondrial genomes revealed significant differences among functional regions in terms of length, nucleotide composition, and skewness patterns (Table S3). The total lengths of the mitochondrial genome exhibited substantial variation (ranging from *Camptocladius stercorarius* at 15,499 bp to *Heterotrissocladius* sp. 1XL at 19,283 bp), whereas the lengths of the PCG region were relatively conserved (ranging from *Parametriocnemus scotti* at 11,181 bp to *Corynoneura isigaheius* at 11,277 bp). The A + T contents of the PCG region (ranging from *Eukiefferiella yasunoi* at 73.69% to *Tvetenia tamaflava* at 81.57%) were markedly higher than the GC contents, with consistently negative AT-skew values, indicating a predominance of thymine bases. The GC-skew was generally negative, although slight positive values were observed in some taxa, suggesting possible interspecific variation.

Notably, the nucleotide composition across the three codon positions displayed a clear gradient; the third codon position exhibited the highest A + T content (range from *Parakiefferiella bathophila* 82.67% to *Compterosmittia nerius* 94.78%) and negative GC-skew, whereas the first position showed a positive GC-skew, reflecting strong site-specific selection pressures in codon usage [67].

The tRNA gene region (ranging from *Brillia bifida* at 1461 bp to *Parametriocnemus scotti* at 1558 bp) demonstrated distinct compositional features, with both the AT-skew and GC-skew being positive (A + T contents ranged from *Compteromesa* sp. 1XL at 76.07% to *Heleniella nebulosa* at 85.79%), indicating an enrichment of adenine and guanine bases. In ribosomal RNA genes, both 16S rRNA (ranging from *Parametriocnemus scotti* at 1302 bp to *Heterotrissocladius* sp. 1XL at 1776 bp) and 12S rRNA (ranging from *Heterotrissocladius* sp. 1XL at 698 bp to *Heleniella nebulosa* at 899 bp) exhibited a predominantly positive GC-skew and negative AT-skew, suggesting similar base usage biases. However, their A + T contents remained high (16S rRNA results ranged from *Brillia flavifrons* at 81.48% to *Heterotrissocladius* sp. 1XL at 90.26%; 12S rRNA results ranged from *Compteromesa* sp. 1XL at 77.93% to *Heleniella nebulosa* at 87.43%), far exceeding the GC content.

The CR displayed the most pronounced variability, with lengths (ranging from *Parakiefferiella* sp. 2XL at 73 bp to *Parakiefferiella viktana* at 2015 bp) and A + T contents (ranging from *Parametriocnemus scotti* at 72.46% to *Parakiefferiella* sp. 2XL at 100%) surpassing all other functional regions; in some taxa, the A + T content reached 100%. AT-skew and GC-skew values in this region fluctuated greatly among species, consistent with the CR being the most rapidly evolving region of the mitochondrial genome [68].

**Figure 2:**
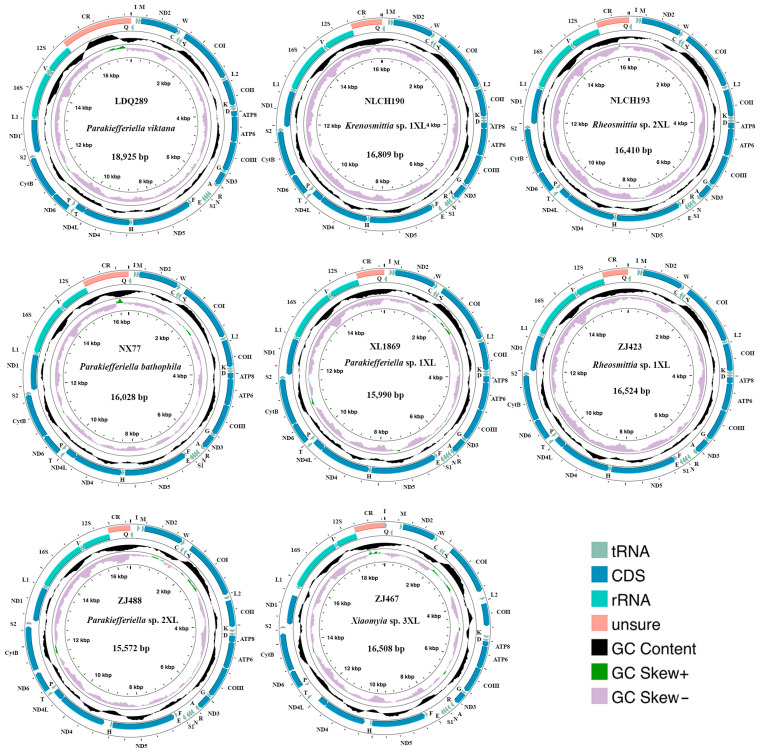
The mitogenome map illustrates the mitochondrial genomes of eight samples exhibiting tRNA gene rearrangements. *Xiaomyia* sp. 3XL showed positional exchange between tRNA-Ala and tRNA-Arg, while the remaining seven specimens exhibited tRNA-Ile/tRNA-Gln swaps. The transcription direction is indicated by arrows. Standardized abbreviations denote PCGs and rRNAs, while tRNAs are represented by single-letter codes. Distinct color schemes differentiate gene functional categories.

Particularly noteworthy was the detection of tRNA gene rearrangements in multiple samples. In sample *Xiaomyia* sp. 3XL, tRNA-Ala and tRNA-Arg had exchanged genomic positions, while in *Krenosmittia* sp. 1XL and six other samples, tRNA-Ile and tRNA-Gln were swapped (Figure 2). These findings not only confirm the dynamic nature of tRNA gene rearrangements during mitochondrial genome evolution but also reveal the substantial structural plasticity and complexity, offering new insights into the structure–function relationship of the mitochondrial genome [69,70].

### 3.2. Codon Usage

**Figure 3:**
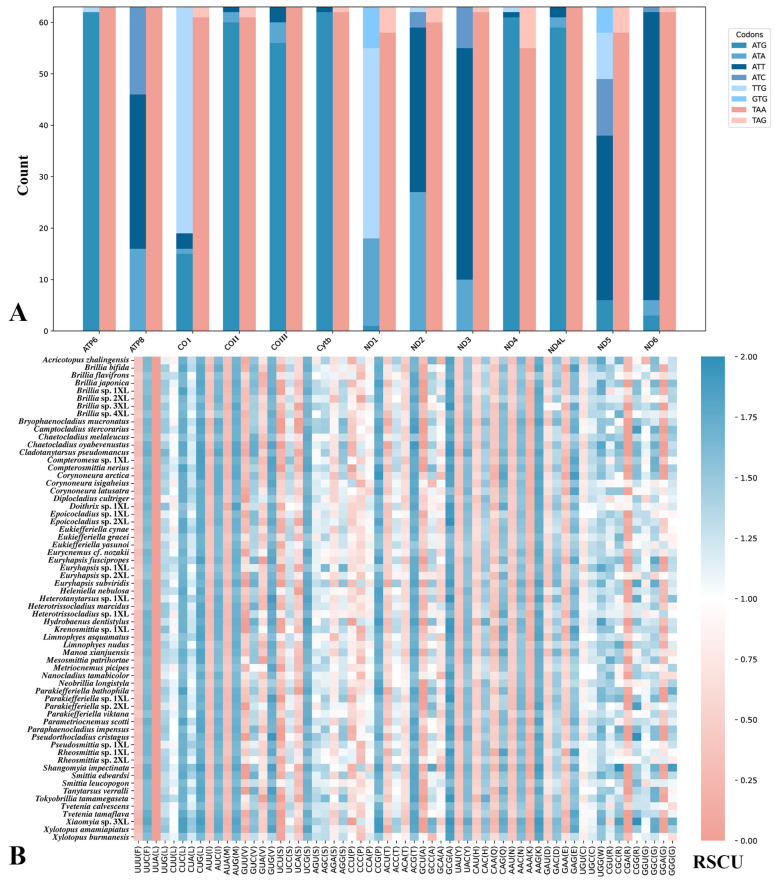
Codon usage patterns in 63 samples. (**A**) Start and stop codon distribution across PCGs. The x-axis represents the 13 PCGs, while the y-axis shows the number of different codons. The varying shades of blue indicate the start codons, and the two shades of red represent the stop codons. (**B**) RSCU values for all codons in 63 samples.

Analysis of all mitochondrial PCGs revealed significant and highly conserved codon usage biases (Figure 3). Among synonymous codons, UUA (Leu), UCU (Ser), UUU (Phe), and UAU (Tyr) showed strong preferences, with RSCU values generally exceeding 1.5 and reaching up to 3.6; UUA was particularly prominent (3.0–3.7). Moderate preferences were observed for GCU (Ala), GAU (Asp), GAA (Glu), GGU (Gly), and GUU (Val) (RSCU: 1.2–1.9). In contrast, codons such as UAC (Tyr), UCC (Ser), AGG (Ser), CGG (Arg), CAG (Gln), and AGC (Ser) were used infrequently (RSCU < 0.6), and stop codons UAA and UAG also showed low usage (RSCU: 0.1–0.9). The high consistency in codon usage patterns among all samples indicates that mitochondrial genomes are under strong evolutionary constraints, maintaining stable preferences over time [71,72]. The distribution of start and stop codons further highlights adaptive features of mitochondrial translation. ATG was the predominant start codon in most genes (e.g., *ATP6*, *COII*, *Cytb*), whereas ATA and ATT were more frequent in *ND2*, *ND3*, *ATP8*, and *ND6*; rare alternative start codons (TTG, GTG) occurred mainly in *COI*, *ND1*, and *ND5*. For stop codons, TAA dominated across all PCGs, while TAG appeared only sporadically, particularly in *COI*, *COII*, *ND1*, and *ND6*. This dual bias in synonymous codon usage and start and stop codon preference likely reflects evolutionary optimization for accuracy and efficiency in translation initiation and termination, as well as gene-specific regulatory requirements or lineage-specific selective pressures, ultimately influencing mitochondrial protein synthesis efficiency and respiratory chain performance [73,74].

### 3.3. Substitution Rates and Nucleotide Diversity

**Figure 4:**
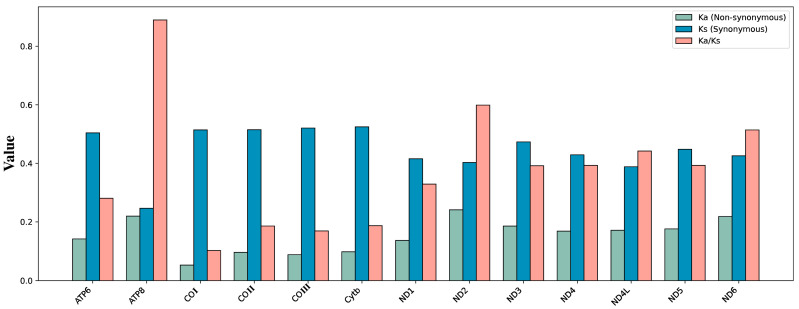
The evolutionary rates of the 13 PCGs in 63 samples. Ka refers to non-synonymous nucleotide substitutions, Ks refers to synonymous nucleotide substitutions, and Ka/Ks refers to the selection pressure acting on each PCG. The x-axis represents the 13 PCGs, while the y-axis shows the Ka/Ks values.

The ratio of non-synonymous to synonymous substitution rates (Ka/Ks) was significantly less than 1 for all genes (range: 0.10–0.89), indicating predominant purifying selection (Figure 4) [75].

There was a clear gradient of selection pressure among genes. The *COI* gene showed the strongest evolutionary conservation (Ka/Ks = 0.10), underscoring its critical role in maintaining mitochondrial function. Within the *ND* gene family, selection pressures were of moderate intensity (Ka/Ks: 0.33–0.60), with *ND1* being the most conserved (0.33) and *ND2* the least constrained (0.60). *ATP6*, *COII*, *COIII*, and *Cytb* displayed Ka/Ks values between 0.17 and 0.28, indicating strong functional constraints [76].

*ATP8* exhibited a Ka/Ks ratio close to neutrality (0.89), suggesting relatively weak purifying selection. This variation in selection patterns has been found in other insects and reflects adaptive divergence among mitochondrial functional genes and offers important insights into the molecular evolutionary mechanisms of mitochondrial genomes [77].

### 3.4. Heterogeneity Analyses of Mitogenomes

**Figure 5:**
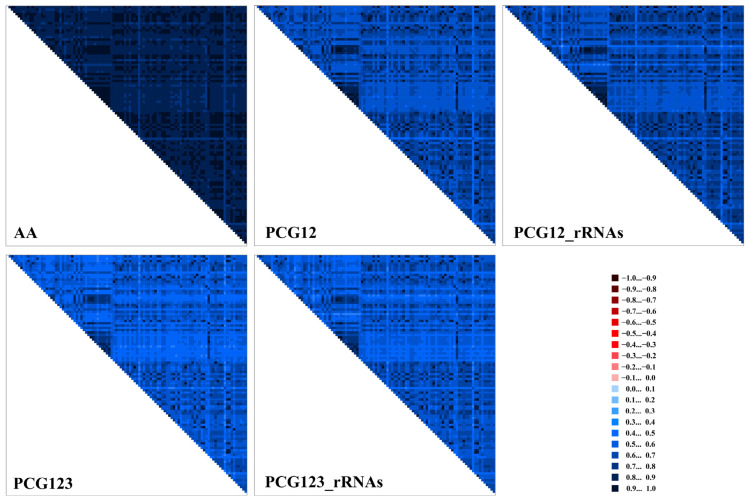
The heterogeneity of the mitogenomes of 104 species from Orthocladiinae, Prodiamesinae and Chironominae based on PCGs, rRNAs, and amino acids. Sequence similarity is visualized using colored blocks, with AliGROOVE scores ranging from −1 (indicating strong heterogeneity between datasets, represented by red) to +1 (indicating weak heterogeneity, represented by blue).

Five datasets with distinct characteristics (AA: 4715 sites; PCG123: 11,181 sites; PCG123_rRNAs: 12,833 sites; PCG12: 7452 sites; PCG12_rRNAs: 9104 sites) were analyzed comparatively (Figures S1–S3). The amino acid dataset exhibited the lowest sequence heterogeneity (Figure 5), largely because synonymous substitutions at third codon positions do not alter the encoded amino acids [78,79].

Heterogeneity levels differed markedly among datasets; PCG123_rRNAs displayed higher heterogeneity than PCG12_rRNAs, and PCG123 was more heterogeneous than PCG12. This pattern can be attributed to two mechanisms: (1) the high degeneracy of third codon positions allows for the accumulation of more neutral mutations, while the first and second positions, which often affect amino acids, are under stronger selective constraint [80,81]; (2) rRNA genes are highly conserved due to their essential roles in ribosomal function [82]. When conserved rRNAs are combined with codon positions, datasets containing third positions (e.g., PCG123_rRNAs) naturally show greater heterogeneity than those containing only conserved positions (e.g., PCG12_rRNAs).

These findings highlight differences in selection pressures across functional regions of the mitochondrial genome and provide a basis for selecting appropriate datasets for phylogenetic analyses [1].

### 3.5. Phylogenetic Analysis

The phylogenetic history of Orthocladiinae has been contentious. While early morphological studies supported its monophyly, its relationship with Prodiamesinae remained unresolved [24,83]. Molecular phylogenetic studies based on multiple gene markers confirmed the monophyly of Orthocladiinae but proposed the reclassification of *Propsilocerus* from its basal position to Prodiamesinae [28]. Subsequent mitochondrial genome analyses further complicated the issue by recovering Prodiamesinae nested within the basal branch of Orthocladiinae [50]. These controversies underscore the necessity of employing genome-scale data to reliably resolve these deep phylogenetic relationships.

**Figure 6:**
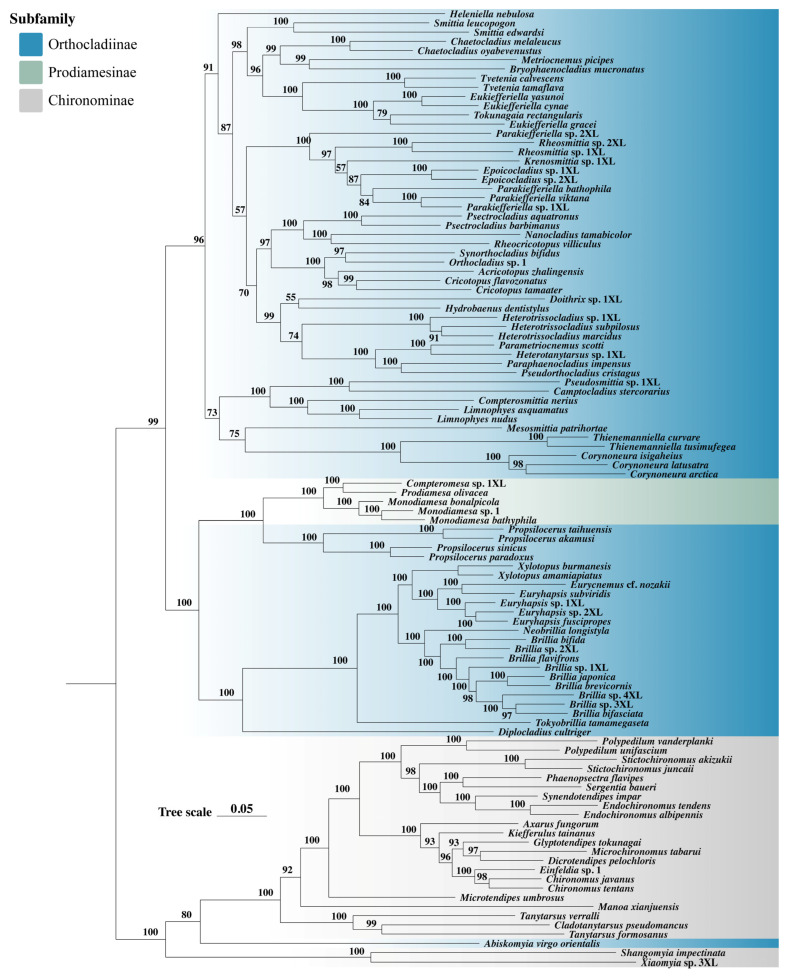
Phylogenetic tree of 104 samples reconstructed from the AA dataset using maximum likelihood (ML) methods. Node supports are indicated by bootstrap values (BS). Nodes with BS < 70 are considered weakly supported and are not labeled.

The phylogenetic results of this study analyses showed that the dataset type had a marked influence on tree topology, particularly in resolving relationships among non-basal lineages within Orthocladiinae. Relationships among non-basal branches differed notably across datasets. For example, the phylogenetic positions of *Hydrobaenus dentistylus* varied substantially. For Prodiamesinae and the basal branch of Orthocladiinae (including *Propsilocerus* and the *Brillia* generic complex), all datasets produced highly consistent topologies (support values ≥ 95, Figure 6). Prodiamesinae was nested within Orthocladiinae and formed a monophyletic group with *Propsilocerus*, consistent with the results based on single-copy ortholog dataset (Unpublished) and the previous study [50].

The ingroup taxon *Abiskomyia virgo orientalis* also exhibited inconsistent placements, clustering within Chironominae in all datasets. Combined with the findings of recent study [41], this suggests possible misidentification of this specimen. In the phylogenetic tree, Chironominae forms a monophyletic group after the exclusion of *Abiskomyia virgo orientalis*. Multiple distinct clades can be clearly recognized within this subfamily. Specifically, six genera (*Polypedilum*, *Stictochironomus*, *Phaenopsectra*, *Sergentia*, *Synendotendipes*, and *Endochironomus*) cluster together to form a monophyletic group, which in turn combines with another monophyletic clade comprising *Axarus*, *Kiefferulus*, *Glyptotendipes*, *Microchironomus*, *Dicrotendipes*, *Einfeldia*, and *Chironomus* to form a larger monophyletic assemblage.

Notably, *Shangomyia* and *Xiaomyia* form a distinct evolutionary branch, constituting a monophyletic group, which establishes Xiaomyiini as a unique clade within the Chironominae. Its distinctiveness is strongly supported by both morphological and molecular evidence. Phylogenetic analyses indicate that these two genera together form the core monophyletic lineage of this tribe. Morphologically, adults exhibit features such as the absence of combs at the tibial apex, an elongated costa, a leg ratio greater than 1.5, and complete fusion of the inferior volsella with the gonocoxite [84]. The larvae possess a specialized ribbed and spinulose plate-like lobe on the ventral side of the mandible, which serves as a key synapomorphy for the tribe, while the pupal exuviae also display distinct morphological characteristics [85]. These autapomorphic morphological traits are consistent with molecular phylogenetic results, collectively confirming that Xiaomyiini represents an independent and early-diverging evolutionary unit. This not only reflects its specific adaptation to freshwater habitats in the Oriental region but also provides an important foundation for future taxonomic revisions and evolutionary studies.

The amino acid dataset, affected by amino acid substitution saturation, yielded shorter branch lengths but more stable topologies, whereas datasets including third codon positions produced longer branches. These results indicate that the mitochondrial genome, being a single genetic locus, may present a biased picture of evolutionary relationships, particularly in cases where introgression or hybridization events have occurred [86]. Future research should integrate nuclear gene data and apply population genomic approaches for a more comprehensive understanding of complex phylogenetic relationships because nuclear genomes represent a broader genetic background, encompassing numerous genes from different chromosomes, providing a more robust dataset for phylogenetic inference [87].

## 4. Conclusions

Based on a systematic analysis of 104 samples, this study elucidates the evolutionary characteristics of mitochondrial genomes in the Orthocladiinae *sensu lato* and Chironominae branch. By sequencing the mitochondrial genomes of 63 newly identified species and reporting genomic data for the rare *Xiaomyia* and *Shangomyia* for the first time, we have substantially expanded the molecular resource base for these groups. The results reveal structural variations, such as tRNA gene rearrangements, and molecular evolutionary patterns characterized by marked nucleotide composition bias, codon usage preferences, and intergenic differences in selective pressure. Distinct evolutionary dynamics were identified among functional regions (PCGs, rRNAs, tRNAs, and the CR). Heterogeneity analyses indicate that amino acid datasets possess a relative advantage for reconstructing deep-level phylogenies, whereas different dataset types exhibit notable limitations in resolving relationships among non-basal clades, providing important guidance for dataset selection in future research. Despite certain inherent limitations, mitochondrial genomes remain a valuable molecular marker for species identification and phylogenetic inference. Moreover, the comprehensive mitochondrial dataset established here offers a valuable resource for rapid species identification and its practical application in aquatic ecological monitoring. Based on the present findings, future studies should prioritize the integration of multi-omics datasets to disentangle complex evolutionary histories and taxonomic relationships.

## Data Availability

All new mitogenomes are deposited in GenBank of NCBI under the accession numbers PX067949–PX06800 and PV994456–PV994466.

## Supplementary Materials

The following supporting information can be downloaded at https://www.mdpi.com/article/10.3390/biology14091178/s1: Table S1. Sample information and collection metadata for 63 newly sequenced species in this study. Table S2. Detailed accession number information of all sequences. Table S3. Nucleotide composition of mitogenomes of 63 newly sequenced species. Figure S1. Phylogenetic tree based on PCG123 dataset. Figure S2. Phylogenetic tree based on PCG123_rRNAs dataset. Figure S3. Phylogenetic tree based on PCG12 dataset. Figure S4. Phylogenetic tree based on PCG12_rRNAs dataset.
