# Supplementary material for: Mitogenomic Insights into Orthocladiinae (Diptera: Chironomidae): Structural Diversity and Phylogenetic Implications
Source: Biology (Basel). 2025 Sep 2;14(9):1178. doi: 10.3390/biology14091178 (PMC12467726; doi:10.3390/biology14091178)
Supplement: Supplementary file 1 [file biology-14-01178-s001.zip › Figure S4.pdf]

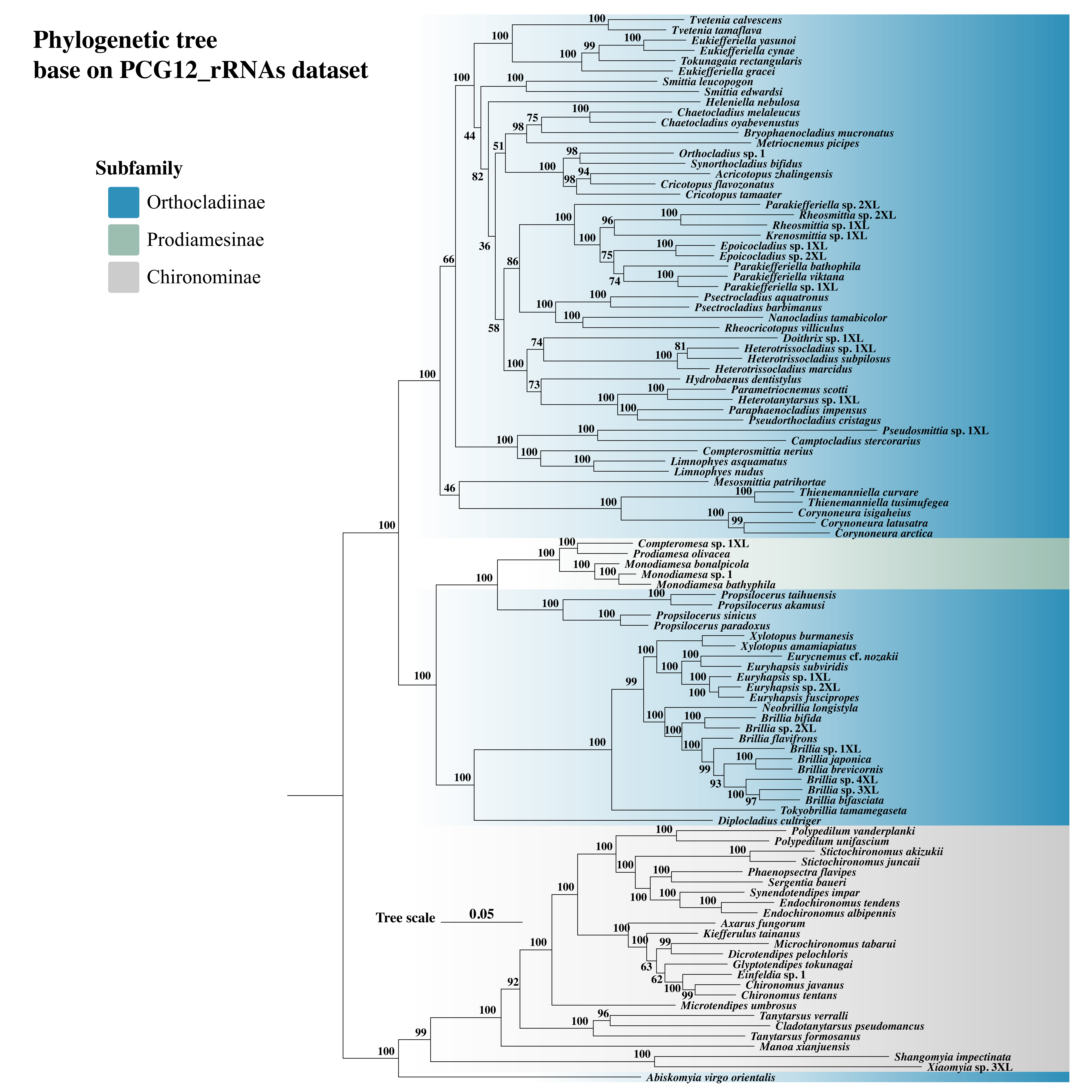

Phylogenetic tree
base on PCG12_rRNAs dataset
Subfamily
Orthocladiinae
Prodiamesinae
Chironominae
Tree scale 0.05
Tvetenia calvescens
Tvetenia tamaflava
Eukiefferiella yasunoi
Eukiefferiella cynae
Tokunagaia rectangularis
Eukiefferiella gracei
Smittia leucopogon
Smittia edwardsi
Heleniella nebulosa
Chaetocladius melaleucus
Chaetocladius oyabevenustus
Bryophaenocladius mucronatus
Metriocnemus picipes
Orthocladius sp. 1
Synorthocladius bifidus
Acricotopus zhalingensis
Cricotopus flavozonatus
Cricotopus tamaater
Parakiefferiella sp. 2XL
Rheosmittia sp. 2XL
Rheosmittia sp. 1XL
Krenosmittia sp. 1XL
Epoicocladius sp. 1XL
Epoicocladius sp. 2XL
Parakiefferiella bathophila
Parakiefferiella viktana
Parakiefferiella sp. 1XL
Psectrocladius aquatronus
Psectrocladius barbimanus
Nanocladius tamabicolor
Rheocricotopus villiculus
Doithrix sp. 1XL
Heterotrissocladius sp. 1XL
Heterotrissocladius subpilosus
Heterotrissocladius marcidus
Hydrobaenus dentistylus
Parametriocnemus scotti
Heterotanytarsus sp. 1XL
Paraphaenocladius impensus
Pseudorthocladius cristagus
Pseudosmittia sp. 1XL
Camptocladius stercorarius
Compterosmittia nerius
Limnophyes asquamatus
Limnophyes nudus
Mesosmittia patrihortae
Thienemanniella curvare
Thienemanniella tusimufegea
Corynoneura isigaheius
Corynoneura latusatra
Corynoneura arctica
Compteromesa sp. 1XL
Prodiamesa olivacea
Monodiamesa bonalpicola
Monodiamesa sp. 1
Monodiamesa bathyphila
Propsilocerus taihuensis
Propsilocerus akamusi
Propsilocerus sinicus
Propsilocerus paradoxus
Xylotopus burmanesis
Xylotopus amamiapiatus
Eurycnemus cf. nozakii
Euryhapsis subviridis
Euryhapsis sp. 1XL
Euryhapsis sp. 2XL
Euryhapsis fuscipropes
Neobrillia longistyla
Brillia bifida
Brillia sp. 2XL
Brillia flavifrons
Brillia sp. 1XL
Brillia japonica
Brillia brevicornis
Brillia sp. 4XL
Brillia sp. 3XL
Brillia bifasciata
Tokyobrillia tamamegaseta
Diplocladius cultriger
Polypedilum vanderplanki
Polypedilum unifascium
Stictochironomus akizukii
Stictochironomus juncaii
Phaenopsectra flavipes
Sergentia baueri
Synendotendipes impar
Endochironomus tendens
Endochironomus albipennis
Axarus fungorum
Kiefferulus tainanus
Microchironomus tabarui
Dicrotendipes pelochloris
Glyptotendipes tokunagai
Einfeldia sp. 1
Chironomus javanus
Chironomus tentans
Microtendipes umbrosus
Tanytarsus verralli
Cladotanytarsus pseudomancus
Tanytarsus formosanus
Manoa xianjuensis
Shangomyia impectinata
Xiaomyia sp. 3XL
Abiskomyia virgo orientalis
