# Supplementary material for: Mitogenomic Insights into Orthocladiinae (Diptera: Chironomidae): Structural Diversity and Phylogenetic Implications
Source: Biology (Basel). 2025 Sep 2;14(9):1178. doi: 10.3390/biology14091178 (PMC12467726; doi:10.3390/biology14091178)
Supplement: Supplementary file 1 [file biology-14-01178-s001.zip › Table S1.pdf]

Sample Information and Collection Metadata for 63 Newly Sequenced Species in this Study

Sample ID	Subfamily	Genus	Species	dev_stage	sex	Collectors	Collection Date	Collection Date Accuracy	Country/Ocean	State/Province	Region	Lat	Lon	Elev	Sampling Protocol	Deposition
CHMIT17	Chironominae	*Manoa*	*Manoa xianjuensis*	adult	male	Xin Qi	24-May-2016	1	China	Zhejiang	Taizhou	28.6327	120.5761	190m	Sweep net	Shanghai Ocean University
XL467	Chironominae	*Xiaomyia*	*Xiaomyia* sp. 3XL	adult	male	Chao Song	25-Apr-2015	1	China	Guizhou	Kaili	27.0434	108.4129	592m	Sweep net	Shanghai Ocean University
XL1444	Chironominae	*Shangomyia*	*Shangomyia impectinata*	adult	male	Bingjiao Sun	07-Mar-2016	1	China	Hainan	Ledong	18.6927	108.7957	60m	Light trap	Shanghai Ocean University
NX25	Chironominae	*Tanytarsus*	*Tanytarsus verralli*	adult	male	Zhilin Huang	01-Sep-2019	5	China	Ningxia	Wuzhong	37.7370	107.3520	1,378m	Malaise Trap	Shanghai Ocean University
NAM49	Chironominae	*Cladotanytarsus*	*Cladotanytarsus pseudomancus*	adult	male	Xiaolong Lin	02-Dec-2018	1	Namibia	Otjozondjupa	Von Bach Dam Nature Reserve	-22.0109	16.9529	1,390m	Sweep net	Shanghai Ocean University
XL1048	Orthocladiinae	*Brillia*	*Brillia* sp. 1XL	adult	female	Xiaolong Lin	26-May-2018	1	China	Yunnan	Kunming	25.0614	102.3530	1,834m	Sweep net	Shanghai Ocean University
XL1964	Orthocladiinae	*Brillia*	*Brillia* sp. 2XL	adult	male	Xiaolong Lin	16-Jul-2014	1	China	Xizang	Nyingchi	29.1873	93.9954	2,976m	Sweep net	Shanghai Ocean University
XL1895	Orthocladiinae	*Brillia*	*Brillia* sp. 3XL	adult	male	Xiaolong Lin	11-Jul-2014	1	China	Xizang	Nyingchi	29.8035	95.8672	2,865m	Sweep net	Shanghai Ocean University
XL3554	Orthocladiinae	*Brillia*	*Brillia* sp. 4XL	adult	male	Xiaolong Lin	20-May-2018	1	China	Yunnan	Dali Bai Autonomous Prefecture	25.6475	100.1426	2,558m	Light trap	Shanghai Ocean University
CHMIT25	Orthocladiinae	*Brillia*	*Brillia bifida*	adult	male	Xiaolong Lin	10-Sep-2019	1	Italy	Veneto	Trentino-Alto Adige/S dtirol and Friuli Venezia Giulia	46.5408	11.6265	1,851m	Sweep net	Shanghai Ocean University
XL3305	Orthocladiinae	*Brillia*	*Brillia flavifrons*	larva	not applicable	Xinyu Ge	12-Jul-2019	1	China	Sichuan	Xizangan Qiang Autonomous Prefecture of Ngawa	33.1928	103.8940	1,200m	D-net	Shanghai Ocean University
XL1786	Orthocladiinae	*Brillia*	*Brillia japonica*	adult	male	Chao Song	09-Jul-2015	1	China	Hubei	Enshi Tujia and Miao Autonomous Prefecture	29.6686	109.6090	793m	Sweep net	Shanghai Ocean University
XL1620	Orthocladiinae	*Eurycnemus*	*Eurycnemus* cf. *nozakii*	adult	male	Chao Song	12-Jul-2016	1	China	Jilin	Baihe	42.4106	128.1110	800m	Light trap	Shanghai Ocean University
XL75	Orthocladiinae	*Euryhapsis*	*Euryhapsis fuscipropes*	adult	male	Xiaolong Lin	08-Jul-2014	1	China	Xizang	Nyingchi	29.6257	94.3705	2,976m	Sweep net	Shanghai Ocean University
XL3675	Orthocladiinae	*Euryhapsis*	*Euryhapsis subviridis*	adult	male	Chao Song	15-Jul-2016	1	China	Jilin	Yanbian Korean Autonomous Prefecture	43.3247	128.2360	511m	Light trap	Shanghai Ocean University
XL2640	Orthocladiinae	*Euryhapsis*	*Euryhapsis* sp. 1XL	adult	male	Chao Song	08-Jul-2016	1	China	Liaoning	Dandong	40.9939	125.2560	196m	Sweep net	Shanghai Ocean University
TMCH13	Orthocladiinae	*Neobrillia*	*Neobrillia longistyla*	adult	male	Qingyun Wang	09-Jun-2020	7	China	Zhejiang	Hangzhou	30.3240	119.4440	351m	Malaise Trap	Shanghai Ocean University
XL2341	Orthocladiinae	*Tokyobrillia*	*Tokyobrillia tamamegaseta*	adult	male	Kaijian Teng	09-Aug-2019	1	China	Yunnan	Hani-Yi Autonomous Prefecture of Honghe	22.9100	103.7000	2,067m	Light trap	Shanghai Ocean University
NLCH295	Orthocladiinae	*Xylotopus*	*Xylotopus amamiapiatus*	adult	male	Xiaolong Lin	30-Aug-2020	1	China	Hunan	Chenzhou	24.9850	112.9140	677m	Light trap	Shanghai Ocean University
B118	Orthocladiinae	*Xylotopus*	*Xylotopus burmanesis*	adult	male	Hongqu Tang	26-May-2018	1	China	Yunnan	Anning	24.5723	102.2607	2,328m	Sweep net	Shanghai Ocean University
CBL120	Orthocladiinae	*Euryhapsis*	*Euryhapsis* sp. 2XL	adult	male	Lirong Lu	16-Dec-2020	5	China	Guangdong	Shaoguan	24.7220	114.2560	380m	Malaise Trap	Shanghai Ocean University
ZJ854	Orthocladiinae	*Bryophaenocladius*	*Bryophaenocladius mucronatus*	adult	male	Xiaolong Lin	01-Aug-2019	3	China	Zhejiang	Wenzhou	28.3276	120.8774	323m	Malaise Trap	Shanghai Ocean University
CHLA363	Orthocladiinae	*Chaetocladius*	*Chaetocladius melaleucus*	larva	not applicable	Xiaolong Lin	20-May-2018	1	China	Yunnan	Dali Bai Autonomous Prefecture	25.6480	100.1430	2,558m	D-net	Shanghai Ocean University
HSY39	Orthocladiinae	*Diplocladius*	*Diplocladius cultriger*	adult	male	Meixin Lin	10-Feb-2020	9	China	Zhejiang	Wenzhou	28.3044	120.9300	570m	Malaise Trap	Shanghai Ocean University
CHLA831	Orthocladiinae	*Eukiefferiella*	*Eukiefferiella cynae*	larva	not applicable	Xinyu Ge	12-Aug-2020	1	China	Qinghai	Huangnan	35.2680	101.8940	3,138m	D-net	Shanghai Ocean University
XL1762	Orthocladiinae	*Heleniella*	*Heleniella nebulosa*	adult	female	Qiang Wang	12-Jul-2015	1	China	Hubei	Enshi Tujia and Miao Autonomous Prefecture	29.8927	110.0290	1,200m	Light trap	Shanghai Ocean University
CBL108	Orthocladiinae	*Corynoneura*	*Corynoneura latusatra*	adult	male	Lirong Lu	26-Nov-2020	5	China	Guangdong	Shaoguan	24.7210	114.2570	355m	Malaise Trap	Shanghai Ocean University
HSY71	Orthocladiinae	*Corynoneura*	*Corynoneura isigaheius*	adult	male	Meixin Lin	22-Apr-2020	10	China	Zhejiang	Wenzhou	28.3040	120.9300	570m	Malaise Trap	Shanghai Ocean University
XL1499	Orthocladiinae	*Compterosmittia*	*Compterosmittia nerius*	adult	male	Chao Song	02-Mar-2016	1	China	Hainan	Wuzhishan	18.8840	109.6640	640m	Light trap	Shanghai Ocean University
XL1660	Orthocladiinae	*Limnophyes*	*Limnophyes asquamatus*	adult	male	Chao Song	11-Jul-2016	1	China	Jilin	Baihe	42.4106	128.1110	713m	Light trap	Shanghai Ocean University
XL1815	Orthocladiinae	*Nanocladius*	*Nanocladius tamabicolor*	adult	male	Bingjiao Sun	23-Jul-2015	1	China	Sichuan	Leshan	29.5733	103.4750	470m	Light trap	Shanghai Ocean University
XL2479	Orthocladiinae	*Metriocnemus*	*Metriocnemus picipes*	adult	male	Bingjiao Sun	11-Jul-2015	1	China	Hubei	Enshi	30.2833	109.4833	1,705m	Sweep net	Shanghai Ocean University
XL2907	Orthocladiinae	*Heterotrissocladius*	*Heterotrissocladius marcidus*	larva	not applicable	Chao Song	07-Sep-2014	1	China	Liaoning	Liaoyang	41.2667	123.1833	50m	D-net	Shanghai Ocean University
XL2958	Orthocladiinae	*Camptocladius*	*Camptocladius stercorarius*	adult	male	Chao Song	17-Jul-2016	1	China	Heilongjiang	Mudanjiang	45.3022	129.7700	215m	Sweep net	Shanghai Ocean University
XL3011	Orthocladiinae	*Pseudorthocladius*	*Pseudorthocladius cristagus*	adult	male	Fanqing Kong	12-Apr-2011	1	China	Zhejiang	Taizhou	29.2523	121.0906	1,100m	Sweep net	Shanghai Ocean University
XL3462	Orthocladiinae	*Tvetenia*	*Tvetenia calvescens*	larva	not applicable	Shuang Qiu	22-Aug-2019	1	China	Jilin	Yanbian Korean Autonomous Prefecture	42.4011	128.1008	714m	D-net	Shanghai Ocean University
XL3693	Orthocladiinae	*Corynoneura*	*Corynoneura arctica*	larva	not applicable	Xiaolong Lin	19-Jul-2014	1	China	Tibet	Xigaze	28.6766	89.6610	4,136m	D-net	Shanghai Ocean University
XL3844	Orthocladiinae	*Limnophyes*	*Limnophyes nudus*	larva	not applicable	Yu Peng	05-Sep-2020	1	China	Tibet	Naqu	31.7128	92.0582	4,628m	D-net	Shanghai Ocean University
ZJ812	Orthocladiinae	*Mesosmittia*	*Mesosmittia patrihortae*	adult	male	Xiaolong Lin	16-Apr-2019	5	China	Zhejiang	Wenzhou	28.3280	120.8770	323m	Malaise Trap	Shanghai Ocean University
XL4306	Orthocladiinae	*Acricotopus*	*Acricotopus zhalingensis*	larva	not applicable	Enlou Zhang	03-Sep-2019	1	China	Tibet	Nagqu	31.8710	87.5860	4,470m	D-net	Shanghai Ocean University
QLF09	Orthocladiinae	*Chaetocladius*	*Chaetocladius oyabevenustus*	adult	male	Rui Guo	15-Feb-2020	10	China	Zhejiang	Hangzhou	30.2741	120.1551	45m	Malaise Trap	Shanghai Ocean University
LSHP560	Orthocladiinae	*Doithrix*	*Doithrix* sp. 1XL	adult	male	Shenggui Zhao	25-Mar-2021	5	China	Guangxi	Guilin	25.5590	109.9400	1,330m	Malaise Trap	Shanghai Ocean University
XL3846	Orthocladiinae	*Eukiefferiella*	*Eukiefferiella gracei*	larva	not applicable	Yu Peng	01-Sep-2020	1	China	Tibet	Naqu	31.3231	89.4370	4,973m	D-net	Shanghai Ocean University
CHLA828	Orthocladiinae	*Eukiefferiella*	*Eukiefferiella yasunoi*	larva	not applicable	Xinyu Ge	12-Aug-2020	1	China	Qinghai	Huangnan	35.2680	101.8940	3,135m	D-net	Shanghai Ocean University
XL1433	Orthocladiinae	*Heterotanytarsus*	*Heterotanytarsus* sp. 1XL	adult	male	Bingjiao Sun	03-Mar-2016	1	China	Hainan	Wuzhishan	18.8927	109.6838	870m	Light trap	Shanghai Ocean University
XL1264	Orthocladiinae	*Heterotrissocladius*	*Heterotrissocladius* sp. 1XL	adult	male	Xiaolong Lin	24-May-2018	1	China	Yunnan	Nujiang of the Lisu Autonomous Prefecture	26.5897	99.0214	3,789m	Light trap	Shanghai Ocean University
XL2036	Orthocladiinae	*Hydrobaenus*	*Hydrobaenus dentistylus*	adult	male	Xiaolong Lin	09-Mar-2019	1	China	Tianjin	Ninghe	39.4243	117.5495	2m	Sweep net	Shanghai Ocean University
NLCH190	Orthocladiinae	*Krenosmittia*	*Krenosmittia* sp. 1XL	adult	male	Xiaolong Lin	27-Aug-2020	2	China	Guangdong	Shaoguan	24.9280	113.0180	1,020m	Light trap	Shanghai Ocean University
LDQ289	Orthocladiinae	*Parakiefferiella*	*Parakiefferiella viktana*	adult	female	Xiaolong Lin	07-Oct-2021	1	China	Tibet	Lhasa	29.7030	92.3360	4,497m	Sweep net	Shanghai Ocean University
NAM19	Orthocladiinae	*Parametriocnemus*	*Parametriocnemus scotti*	adult	male	Xiaolong Lin	01-Dec-2018	1	Namibia	Mariental	Hardap	-24.2625	16.2280	1,400m	Sweep net	Shanghai Ocean University
NAM86	Orthocladiinae	*Pseudosmittia*	*Pseudosmittia* sp. 1XL	adult	male	Xiaolong Lin	03-Dec-2018	1	Namibia	Khomas	Windhoek	-22.5290	17.0010	1,630m	Sweep net	Shanghai Ocean University
ZJ423	Orthocladiinae	*Rheosmittia*	*Rheosmittia* sp. 1XL	larva	not applicable	Haijun Yu	29-Jul-2019	1	China	Zhejiang	Lishui	28.3937	118.8450	485m	D-net	Shanghai Ocean University
XL2667	Orthocladiinae	*Smittia*	*Smittia edwardsi*	adult	female	Chao Song	06-Jul-2016	1	China	Liaoning	Dandong	40.7313	124.7840	347m	Light trap	Shanghai Ocean University
XL2049	Orthocladiinae	*Smittia*	*Smittia leucopogon*	adult	male	Haijun Yu	26-Jan-2019	4	China	Guizhou	Qiandongnan Miao and Dong Autonomous Prefecture	26.4057	108.0786	847m	Malaise Trap	Shanghai Ocean University
XL989	Orthocladiinae	*Tvetenia*	*Tvetenia tamaflava*	adult	male	Xiaolong Lin	21-May-2018	1	China	Yunnan	Dali	25.6431	100.0370	1,759m	Light trap	Shanghai Ocean University
NX77	Orthocladiinae	*Parakiefferiella*	*Parakiefferiella bathophila*	adult	male	Zhilin Huang	22-Oct-2019	5	China	Ningxia	Wuzhong	37.7370	107.3520	1,378m	Malaise Trap	Shanghai Ocean University
NAM83	Orthocladiinae	*Paraphaenocladius*	*Paraphaenocladius impensus*	adult	male	Xiaolong Lin	03-Dec-2018	1	Namibia	Khomas	Windhoek	-22.5290	17.0010	1,630m	Sweep net	Shanghai Ocean University
XL3989	Orthocladiinae	*Epoicocladius*	*Epoicocladius* sp. 2XL	larva	not applicable	Qingbo Huo	24-Aug-2020	1	China	Hunan	Shaoyang	26.3998	111.0030	772m	D-net	Shanghai Ocean University
ZJ488	Orthocladiinae	*Parakiefferiella*	*Parakiefferiella* sp. 2XL	larva	not applicable	Haijun Yu	28-Jul-2019	1	China	Zhejiang	Lishui	28.3910	118.8450	484m	D-net	Shanghai Ocean University
XL1065	Orthocladiinae	*Epoicocladius*	*Epoicocladius* sp. 1XL	adult	male	Xiaolong Lin	26-May-2018	1	China	Yunnan	Kunming	25.2078	102.8273	1,987m	Light trap	Shanghai Ocean University
XL1869	Orthocladiinae	*Parakiefferiella*	*Parakiefferiella* sp. 1XL	adult	male	Yike Han	12-Jun-2015	1	China	Xinjiang	Ili Kazak Autonomous Prefecture	43.9778	81.5276	790m	Light trap	Shanghai Ocean University
NLCH193	Orthocladiinae	*Rheosmittia*	*Rheosmittia* sp. 2XL	adult	male	Xiaolong Lin	27-Aug-2020	2	China	Guangdong	Shaoguan	24.9280	113.0180	1,020m	Light trap	Shanghai Ocean University
CBL122	Prodiamesinae	*Compteromesa*	*Compteromesa* sp. 1XL	adult	male	Lirong Lu	16-Dec-2020	5	China	Guangdong	Shaoguan	24.7220	114.2560	380m	Malaise Trap	Shanghai Ocean University
