# Supplementary material for: Mitogenomic Insights into Orthocladiinae (Diptera: Chironomidae): Structural Diversity and Phylogenetic Implications
Source: Biology (Basel). 2025 Sep 2;14(9):1178. doi: 10.3390/biology14091178 (PMC12467726; doi:10.3390/biology14091178)
Supplement: Supplementary file 1 [file biology-14-01178-s001.zip › Table S2.pdf]

Detail accession number information of all sequences						
序号	Subfamily	Tribe	Genus	Species	Reference	GeneBank accession number
1	Chironominae	Chironomini	*Axarus*	*Axarus fungorum*	[48]	ON099430
2	Chironominae	Chironomini	*Chironomus*	*Chironomus javanus*	[39]	ON975025
3	Chironominae	Chironomini	*Chironomus*	*Chironomus tentans*	[39]	ON975031
4	Chironominae	Chironomini	*Dicrotendipes*	*Dicrotendipes pelochloris*	[42]	ON838257
5	Chironominae	Chironomini	*Einfeldia*	*Einfeldia* sp. 1	[42]	ON943041
6	Chironominae	Chironomini	*Endochironomus*	*Endochironomus albipennis*	[46]	OP950227
7	Chironominae	Chironomini	*Endochironomus*	*Endochironomus tendens*	[46]	OP950219
8	Chironominae	Chironomini	*Glyptotendipes*	*Glyptotendipes tokunagai*	[42]	MZ747091
9	Chironominae	Chironomini	*Kiefferulus*	*Kiefferulus tainanus*	[42]	ON838256
10	Chironominae	Chironomini	*Microchironomus*	*Microchironomus tabarui*	[43]	MZ261913
11	Chironominae	Chironomini	*Microtendipes*	*Microtendipes umbrosus*	[47]	MZ981734
12	Chironominae	Chironomini	*Phaenopsectra*	*Phaenopsectra flavipes*	[46]	OP950216
13	Chironominae	Chironomini	*Polypedilum*	*Polypedilum unifascium*	[45]	MW677959
14	Chironominae	Chironomini	*Polypedilum*	*Polypedilum vanderplanki*	[45]	KT251040
15	Chironominae	Chironomini	*Sergentia*	*Sergentia baueri*	[46]	OP950220
16	Chironominae	Chironomini	*Stictochironomus*	*Stictochironomus akizukii*	[46]	OP950218
17	Chironominae	Chironomini	*Stictochironomus*	*Stictochironomus juncaii*	[46]	OP950226
18	Chironominae	Chironomini	*Synendotendipes*	*Synendotendipes impar*	[46]	OP950223
19	Chironominae	Pseudochironomini	*Manoa*	*Manoa xianjuensis*	This study	PX067955
20	Chironominae	Tanytarsini	*Cladotanytarsus*	*Cladotanytarsus pseudomancus*	This study	PX067961
21	Chironominae	Tanytarsini	*Tanytarsus*	*Tanytarsus formosanus*	[42]	ON838255
22	Chironominae	Tanytarsini	*Tanytarsus*	*Tanytarsus verralli*	This study	PV994466
23	Chironominae	Xiaomyini	*Shangomyia*	*Shangomyia impectinata*	This study	PX067975
24	Chironominae	Xiaomyini	*Xiaomyia*	*Xiaomyia* sp. 3XL	This study	PX067970
25	Orthocladiinae	/	*Abiskomyia*	*Abiskomyia virgo orientalis*	[41]	PP761354
26	Orthocladiinae	/	*Acricotopus*	*Acricotopus zhalingensis*	This study	PX067999
27	Orthocladiinae	/	*Brillia*	*Brillia bifasciata*	[50]	MZ424310
28	Orthocladiinae	/	*Brillia*	*Brillia bifida*	This study	PX067956
29	Orthocladiinae	/	*Brillia*	*Brillia brevicornis*	[50]	MZ424311
30	Orthocladiinae	/	*Brillia*	*Brillia flavifrons*	This study	PX067992
31	Orthocladiinae	/	*Brillia*	*Brillia japonica*	This study	PX067980
32	Orthocladiinae	/	*Brillia*	*Brillia* sp. 1XL	This study	PX067971
33	Orthocladiinae	/	*Brillia*	*Brillia* sp. 2XL	This study	PX067983
34	Orthocladiinae	/	*Brillia*	*Brillia* sp. 3XL	This study	PX067982
35	Orthocladiinae	/	*Brillia*	*Brillia* sp. 4XL	This study	PX067993
36	Orthocladiinae	/	*Bryophaenocladius*	*Bryophaenocladius mucronatus*	This study	PX068000
37	Orthocladiinae	/	*Camptocladius*	*Camptocladius stercorarius*	This study	PX067990
38	Orthocladiinae	/	*Chaetocladius*	*Chaetocladius melaleucus*	This study	PX067953
39	Orthocladiinae	/	*Chaetocladius*	*Chaetocladius oyabevenustus*	This study	PX067967
40	Orthocladiinae	/	*Compterosmittia*	*Compterosmittia nerius*	This study	PX067976
41	Orthocladiinae	/	*Corynoneura*	*Corynoneura arctica*	This study	PX067995
42	Orthocladiinae	/	*Corynoneura*	*Corynoneura isigaheius*	This study	PX067958
43	Orthocladiinae	/	*Corynoneura*	*Corynoneura latusatra*	This study	PX067950
44	Orthocladiinae	/	*Cricotopus*	*Cricotopus flavozonatus*	[37]	OP006247
45	Orthocladiinae	/	*Cricotopus*	*Cricotopus tamaater*	[50]	MW837768
46	Orthocladiinae	/	*Diplocladius*	*Diplocladius cultriger*	This study	PX067957
47	Orthocladiinae	/	*Doithrix*	*Doithrix* sp. 1XL	This study	PX067959
48	Orthocladiinae	/	*Epoicocladius*	*Epoicocladius* sp. 1XL	This study	PX067972
49	Orthocladiinae	/	*Epoicocladius*	*Epoicocladius* sp. 2XL	This study	PX067998
50	Orthocladiinae	/	*Eukiefferiella*	*Eukiefferiella cynae*	This study	PV994456
51	Orthocladiinae	/	*Eukiefferiella*	*Eukiefferiella gracei*	This study	PX067997
52	Orthocladiinae	/	*Eukiefferiella*	*Eukiefferiella yasunoi*	This study	PX067954
53	Orthocladiinae	/	*Eurycnemus*	*Eurycnemus* cf. *nozakii*	This study	PX067977
54	Orthocladiinae	/	*Euryhapsis*	*Euryhapsis fuscipropes*	This study	PX067969
55	Orthocladiinae	/	*Euryhapsis*	*Euryhapsis* sp. 1XL	This study	PX067987
56	Orthocladiinae	/	*Euryhapsis*	*Euryhapsis* sp. 2XL	This study	PX067951
57	Orthocladiinae	/	*Euryhapsis*	*Euryhapsis subviridis*	This study	PX067994
58	Orthocladiinae	/	*Heleniella*	*Heleniella nebulosa*	This study	PX067979
59	Orthocladiinae	/	*Heterotanytarsus*	*Heterotanytarsus* sp. 1XL	This study	PX067974
60	Orthocladiinae	/	*Heterotrissocladius*	*Heterotrissocladius marcidus*	This study	PX067989
61	Orthocladiinae	/	*Heterotrissocladius*	*Heterotrissocladius* sp. 1XL	This study	PX067973

62	Orthocladiinae	/	*Heterotrissocladius*	*Heterotrissocladius subpilosus*	[41]	PP761362
63	Orthocladiinae	/	*Hydrobaenus*	*Hydrobaenus dentistylus*	This study	PX067984
64	Orthocladiinae	/	*Krenosmittia*	*Krenosmittia* sp. 1XL	This study	PX067963
65	Orthocladiinae	/	*Limnophyes*	*Limnophyes asquamatus*	This study	PX067978
66	Orthocladiinae	/	*Limnophyes*	*Limnophyes nudus*	This study	PX067996
67	Orthocladiinae	/	*Mesosmittia*	*Mesosmittia patrihortae*	This study	PV994465
68	Orthocladiinae	/	*Metriocnemus*	*Metriocnemus picipes*	This study	PX067986
69	Orthocladiinae	/	*Nanocladius*	*Nanocladius tamabicolor*	This study	PX067981
70	Orthocladiinae	/	*Neobrillia*	*Neobrillia longistyla*	This study	PX067968
71	Orthocladiinae	/	*Orthocladius*	*Orthocladius* sp. 1	[41]	PP761360
72	Orthocladiinae	/	*Parakiefferiella*	*Parakiefferiella bathophila*	This study	PX067966
73	Orthocladiinae	/	*Parakiefferiella*	*Parakiefferiella* sp. 1XL	This study	PV994460
74	Orthocladiinae	/	*Parakiefferiella*	*Parakiefferiella* sp. 2XL	This study	PV994464
75	Orthocladiinae	/	*Parakiefferiella*	*Parakiefferiella viktana*	This study	PV994457
76	Orthocladiinae	/	*Parametriocnemus*	*Parametriocnemus scotti*	This study	PX067960
77	Orthocladiinae	/	*Paraphaenocladius*	*Paraphaenocladius impensus*	This study	PX067962
78	Orthocladiinae	/	*Propsilocerus*	*Propsilocerus akamusi*	[50]	MW846253
79	Orthocladiinae	/	*Propsilocerus*	*Propsilocerus paradoxus*	[50]	MW846254
80	Orthocladiinae	/	*Propsilocerus*	*Propsilocerus sinicus*	[50]	MW846255
81	Orthocladiinae	/	*Propsilocerus*	*Propsilocerus taihuensis*	[50]	MW837766
82	Orthocladiinae	/	*Psectrocladius*	*Psectrocladius aquatronus*	[49]	PV132386
83	Orthocladiinae	/	*Psectrocladius*	*Psectrocladius barbimanus*	[49]	PV132387
84	Orthocladiinae	/	*Pseudorthocladius*	*Pseudorthocladius cristagus*	This study	PX067991
85	Orthocladiinae	/	*Pseudosmittia*	*Pseudosmittia* sp. 1XL	This study	PV994458
86	Orthocladiinae	/	*Rheocricotopus*	*Rheocricotopus villiculus*	[38]	MW373526
87	Orthocladiinae	/	*Rheosmittia*	*Rheosmittia* sp. 1XL	This study	PV994463
88	Orthocladiinae	/	*Rheosmittia*	*Rheosmittia* sp. 2XL	This study	PX067964
89	Orthocladiinae	/	*Smittia*	*Smittia edwardsi*	This study	PX067988
90	Orthocladiinae	/	*Smittia*	*Smittia leucopogon*	This study	PV994461
91	Orthocladiinae	/	*Synorthocladius*	*Synorthocladius bifidus*	[37]	OP006248
92	Orthocladiinae	/	*Thienemanniella*	*Thienemanniella curvare*	[40]	OR333979
93	Orthocladiinae	/	*Thienemanniella*	*Thienemanniella tusimufegea*	[40]	OR333983
94	Orthocladiinae	/	*Tokunagaia*	*Tokunagaia rectangularis*	[41]	PP761367
95	Orthocladiinae	/	*Tokyobrillia*	*Tokyobrillia tamamegaseta*	This study	PX067985
96	Orthocladiinae	/	*Tvetenia*	*Tvetenia calvescens*	This study	PV994462
97	Orthocladiinae	/	*Tvetenia*	*Tvetenia tamaflava*	This study	PV994459
98	Orthocladiinae	/	*Xylotopus*	*Xylotopus amamiapiatus*	This study	PX067965
99	Orthocladiinae	/	*Xylotopus*	*Xylotopus burmanesis*	This study	PX067949
100	Prodiamesinae	/	*Compteromesa*	*Compteromesa* sp. 1XL	This study	PX067952
101	Prodiamesinae	/	*Monodiamesa*	*Monodiamesa bathyphila*	[41]	PP761358
102	Prodiamesinae	/	*Monodiamesa*	*Monodiamesa bonalpicola*	[50]	MW837770
103	Prodiamesinae	/	*Monodiamesa*	*Monodiamesa* sp. 1	[50]	MW837769
104	Prodiamesinae	/	*Prodiamesa*	*Prodiamesa olivacea*	[38]	MW373525
