# Supplementary material for: Mitogenomic Insights into Orthocladiinae (Diptera: Chironomidae): Structural Diversity and Phylogenetic Implications
Source: Biology (Basel). 2025 Sep 2;14(9):1178. doi: 10.3390/biology14091178 (PMC12467726; doi:10.3390/biology14091178)
Supplement: Supplementary file 1 [file biology-14-01178-s001.zip › Table S3.pdf]

Nucleotide composition of mitogenomes of 63 newly sequenced species

Regions	Species	mitogenome	PCGs	1st Codon Position	2nd Codon Position	3rd Codon Position	tRNA	16S rRNA	12S rRNA	CR
Length（bp）	*Acricotopus zhalingensis*	15,827	11,217	3,739	3,739	3,739	1,484	1,404	815	424
	Brillia bifida	15,769	11,202	3,734	3,734	3,734	1,461	1,422	817	690
	Brillia flavifrons	15,610	11,208	3,736	3,736	3,736	1,470	1,377	781	637
	Brillia japonica	16,497	11,217	3,739	3,739	3,739	1,488	1,419	819	717
	Brillia sp. 1XL	15,766	11,211	3,737	3,737	3,737	1,481	1,408	816	538
	Brillia sp. 2XL	15,925	11,217	3,739	3,739	3,739	1,476	1,396	813	767
	Brillia sp. 3XL	16,635	11,211	3,737	3,737	3,737	1,493	1,410	804	664
	Brillia sp. 4XL	16,430	11,238	3,746	3,746	3,746	1,486	1,439	836	952
	Bryophaenocladius mucronatus	15,825	11,232	3,744	3,744	3,744	1,482	1,390	822	736
	Camptocladius stercorarius	15,499	11,223	3,741	3,741	3,741	1,474	1,384	794	317
	Chaetocladius melaleucus	16,512	11,235	3,745	3,745	3,745	1,478	1,400	807	1,101
	Chaetocladius oyabevenustus	16,658	11,217	3,739	3,739	3,739	1,475	1,428	831	1,168
	Cladotanytarsus pseudomancus	17,664	11,205	3,735	3,735	3,735	1,534	1,563	838	1,252
	Compteromesa sp. 1XL	16,130	11,217	3,739	3,739	3,739	1,475	1,389	811	904
	Compterosmittia nerius	16,635	11,241	3,747	3,747	3,747	1,471	1,373	797	855
	Corynoneura arctica	16,841	11,235	3,745	3,745	3,745	1,475	1,437	808	1,153
	Corynoneura isigaheius	16,427	11,277	3,759	3,759	3,759	1,534	1,374	796	1,167
	Corynoneura latusatra	16,785	11,217	3,739	3,739	3,739	1,500	1,407	816	856
	Diplocladius cultriger	16,200	11,253	3,751	3,751	3,751	1,480	1,419	833	1,019
	Doithrix sp. 1XL	16,984	11,223	3,741	3,741	3,741	1,508	1,415	824	929
	Epoicocladius sp. 1XL	16,401	11,223	3,741	3,741	3,741	1,481	1,442	835	1,046
	Epoicocladius sp. 2XL	15,952	11,232	3,744	3,744	3,744	1,486	1,404	832	789
	Eukiefferiella cynae	16,374	11,208	3,736	3,736	3,736	1,491	1,447	834	880
	Eukiefferiella gracei	16,254	11,235	3,745	3,745	3,745	1,517	1,429	818	865
	Eukiefferiella yasunoi	16,871	11,223	3,741	3,741	3,741	1,507	1,393	810	1,628
	Eurycnemus cf. nozakii	17,146	11,223	3,741	3,741	3,741	1,479	1,456	809	1,657
	Euryhapsis fuscipropes	16,311	11,220	3,740	3,740	3,740	1,472	1,378	804	1,175
	Euryhapsis sp. 1XL	15,873	11,238	3,746	3,746	3,746	1,484	1,394	825	770
	Euryhapsis sp. 2XL	16,355	11,259	3,753	3,753	3,753	1,505	1,436	835	987
	Euryhapsis subviridis	16,573	11,211	3,737	3,737	3,737	1,492	1,395	848	1,357
	Heleniella nebulosa	18,032	11,229	3,743	3,743	3,743	1,485	1,697	899	504
	Heterotanytarsus sp. 1XL	15,690	11,205	3,735	3,735	3,735	1,484	1,395	822	286
	Heterotrissocladius marcidus	16,388	11,205	3,735	3,735	3,735	1,493	1,405	814	1,130
	Heterotrissocladius sp. 1XL	19,283	11,214	3,738	3,738	3,738	1,529	1,776	698	921
	Hydrobaenus dentistylus	15,747	11,259	3,753	3,753	3,753	1,481	1,394	827	605
	Krenosmittia sp. 1XL	16,809	11,208	3,736	3,736	3,736	1,490	1,530	822	827
	Limnophyes asquamatus	16,458	11,220	3,740	3,740	3,740	1,509	1,484	864	984
	Limnophyes nudus	16,340	11,229	3,743	3,743	3,743	1,524	1,403	816	911
	Manoa xianjuensis	16,074	11,226	3,742	3,742	3,742	1,479	1,393	808	725
	Mesosmittia patrihortae	16,549	11,214	3,738	3,738	3,738	1,483	1,408	816	716
	Metriocnemus picipes	15,720	11,217	3,739	3,739	3,739	1,472	1,359	806	685
	Nanocladius tamabicolor	15,903	11,265	3,755	3,755	3,755	1,486	1,413	831	587
	Neobrillia longistyla	16,361	11,253	3,751	3,751	3,751	1,500	1,527	847	300
	Parakiefferiella bathophila	16,028	11,211	3,737	3,737	3,737	1,483	1,363	792	821
	Parakiefferiella sp. 1XL	15,990	11,211	3,737	3,737	3,737	1,471	1,368	796	863
	Parakiefferiella sp. 2XL	15,572	11,232	3,744	3,744	3,744	1,492	1,398	813	73
	Parakiefferiella viktana	18,925	11,214	3,738	3,738	3,738	1,505	1,411	802	2,015
	Parametriocnemus scotti	17,371	11,181	3,727	3,727	3,727	1,558	1,302	807	1,906
	Paraphaenocladius impensus	16,708	11,211	3,737	3,737	3,737	1,498	1,473	842	1,063
	Pseudorthocladius cristagus	15,689	11,208	3,736	3,736	3,736	1,486	1,498	820	328
	Pseudosmittia sp. 1XL	16,153	11,199	3,733	3,733	3,733	1,480	1,399	804	858
	Rheosmittia sp. 1XL	16,524	11,244	3,748	3,748	3,748	1,491	1,423	803	578

	Rheosmittia sp. 2XL	16,410	11,214	3,738	3,738	3,738	1,516	1,439	822	1,106
	Shangomyia impectinata	15,622	11,217	3,739	3,739	3,739	1,476	1,380	826	542
	Smittia edwardsi	16,225	11,241	3,747	3,747	3,747	1,478	1,420	832	977
	Smittia leucopogon	16,585	11,223	3,741	3,741	3,741	1,488	1,387	790	1,577
	Tanytarsus verralli	16,030	11,214	3,738	3,738	3,738	1,490	1,382	823	780
	Tokyobrillia tamamegaseta	15,841	11,265	3,755	3,755	3,755	1,477	1,328	787	986
	Tvetenia calvescens	15,867	11,202	3,734	3,734	3,734	1,481	1,371	795	641
	Tvetenia tamaflava	16,376	11,220	3,740	3,740	3,740	1,492	1,373	816	1,190
	Xiaomyia sp. 3XL	16,508	11,214	3,738	3,738	3,738	1,502	1,368	801	802
	Xylotopus amamiapiatus	15,775	11,187	3,729	3,729	3,729	1,548	1,397	812	559
	Xylotopus burmanesis	16,330	11,262	3,754	3,754	3,754	1,462	1,431	827	963
	Acricotopus zhalingensis	76.86	77.20	72.63	70.48	88.50	79.45	85.40	83.19	84.20
	Brillia bifida	79.55	78.63	74.49	71.75	89.65	82.89	86.71	86.41	90.72
	Brillia flavifrons	75.52	76.00	71.56	68.30	88.14	80.34	81.48	79.90	90.42
	Brillia japonica	80.42	78.20	73.17	69.78	91.65	81.25	86.75	85.10	93.86
	Brillia sp. 1XL	77.52	76.42	70.85	68.88	89.53	78.66	84.45	82.11	94.61
	Brillia sp. 2XL	79.3	78.08	72.49	69.39	92.36	82.72	85.17	83.89	89.83
	Brillia sp. 3XL	78.11	75.47	71.11	68.56	86.75	79.84	85.11	83.58	90.06
	Brillia sp. 4XL	79.24	77.97	72.91	69.89	91.11	79.68	84.57	81.70	93.91
	Bryophaenocladius mucronatus	75.78	75.40	70.56	67.93	87.72	77.53	83.74	82.24	91.71
	Camptocladius stercorarius	78.68	77.87	71.80	69.42	92.38	81.61	84.68	83.25	89.59
	Chaetocladius melaleucus	77.61	76.19	71.26	68.64	88.68	78.96	84.50	81.91	85.10
	Chaetocladius oyabevenustus	79.3	77.56	71.64	69.25	91.80	79.66	84.59	82.55	94.01
	Cladotanytarsus pseudomancus	78.01	75.02	71.31	68.54	85.20	82.66	86.18	84.25	92.41
	Compteromesa sp. 1XL	76.14	74.60	68.89	66.40	88.52	76.07	83.15	77.93	92.48
	Compterosmittia nerius	78.53	81.49	76.63	73.07	94.78	79.47	84.20	83.81	92.63
	Corynoneura arctica	78.33	77.00	71.09	69.83	90.09	79.66	85.46	83.42	93.15
	Corynoneura isigaheius	79.02	77.80	72.42	69.75	91.22	80.51	83.41	81.53	91.43
	Corynoneura latusatra	80.11	77.91	72.89	70.41	90.42	81.6	86.85	85.42	90.89
	Diplocladius cultriger	77.62	76.15	70.82	69.58	88.06	78.04	84.71	82.23	90.97
	Doithrix sp. 1XL	79.64	77.09	71.27	70.39	89.60	81.3	86.22	85.92	90.74
	Epoicocladius sp. 1XL	79.06	75.20	70.71	68.96	85.92	79.61	84.05	82.28	93.40
	Epoicocladius sp. 2XL	77.95	78.47	72.34	70.03	93.04	77.73	84.40	82.57	94.04
	Eukiefferiella cynae	79.57	74.84	71.51	68.11	84.90	79.68	84.93	82.85	94.43
	Eukiefferiella gracei	79.27	74.51	69.43	69.30	84.80	82.6	85.86	84.96	92.83
	Eukiefferiella yasunoi	75.54	73.69	69.98	68.00	83.09	78.5	84.64	82.47	79.67
	Eurycnemus cf. nozakii	77.73	78.63	73.95	69.82	92.13	80.32	85.71	83.81	89.74
	Euryhapsis fuscipropes	79.09	77.41	72.16	69.51	90.57	80.43	85.70	84.20	90.38
	Euryhapsis sp. 1XL	75.81	77.82	72.66	69.39	91.41	77.96	84.36	82.18	94.16
	Euryhapsis sp. 2XL	77.12	75.26	70.57	69.00	86.22	79.6	83.57	82.16	92.40
	Euryhapsis subviridis	78.94	77.42	72.43	69.30	90.54	82.37	85.16	84.08	86.15
	Heleniella nebulosa	84.23	76.11	71.76	68.54	88.04	85.79	90.16	87.43	94.05
A+T%	*Heterotanytarsus* sp. 1XL	76.95	75.00	71.82	68.00	85.20	80.26	85.02	84.43	88.81
	Heterotrissocladius marcidus	80.2	74.27	69.86	68.01	84.95	81.31	86.12	84.40	93.01
	Heterotrissocladius sp. 1XL	85.29	76.46	71.92	67.90	89.55	84.63	90.26	85.39	97.39
	Hydrobaenus dentistylus	77.35	74.31	69.60	69.59	83.72	78.8	84.07	82.71	95.54
	Krenosmittia sp. 1XL	80.13	78.43	73.91	71.19	90.20	81.48	87.97	85.40	92.74
	Limnophyes asquamatus	79.85	76.81	72.43	69.20	88.78	82.5	86.46	85.76	92.58
	Limnophyes nudus	79.94	75.76	71.74	68.31	87.23	83.07	87.03	84.44	93.63
	Manoa xianjuensis	76.15	74.15	69.57	67.40	85.49	78.57	84.57	81.68	88.41
	Mesosmittia patrihortae	79.15	76.79	72.82	69.70	87.86	82.67	85.87	82.35	93.99
	Metriocnemus picipes	79.22	78.67	73.14	69.02	93.84	80.64	85.06	84.49	92.55
	Nanocladius tamabicolor	77.96	76.19	70.71	69.11	88.74	80.28	85.00	82.31	91.82
	Neobrillia longistyla	80.12	78.19	73.97	70.79	89.83	82.47	87.69	85.83	96.67
	Parakiefferiella bathophila	75.84	73.87	70.42	68.52	82.67	81.05	84.52	82.83	84.90
	Parakiefferiella sp. 1XL	77.49	77.43	71.00	69.32	91.96	78.93	84.21	83.67	93.51
	Parakiefferiella sp. 2XL	78.6	77.46	73.21	69.97	89.19	82.37	84.76	83.76	100.00
	Parakiefferiella viktana	79.66	75.61	70.81	68.58	87.45	81.26	85.97	84.04	84.91

	Parametriocnemus scotti	76.6	75.88	71.45	68.74	87.45	81.51	83.95	84.01	72.46
	Paraphaenocladius impensus	78.3	75.78	71.01	68.53	87.79	81.11	85.13	85.04	92.76
	Pseudorthocladius cristagus	78.41	77.04	73.09	69.00	89.02	80.42	86.85	84.02	95.73
	Pseudosmittia sp. 1XL	77.45	78.06	71.58	69.58	93.02	79.19	84.27	82.71	93.12
	Rheosmittia sp. 1XL	80.13	78.14	72.98	69.81	91.62	81.15	86.37	84.06	95.33
	Rheosmittia sp. 2XL	79.24	77.14	72.05	68.82	90.56	82.06	85.82	84.31	95.39
	Shangomyia impectinata	76.57	77.65	72.11	69.40	91.45	78.59	84.13	81.36	93.73
	Smittia edwardsi	77.23	77.52	71.75	70.59	90.20	77.94	83.94	81.61	93.24
	Smittia leucopogon	79.57	75.59	71.68	69.63	85.47	79.17	84.57	82.53	92.07
	Tanytarsus verralli	77.84	76.24	73.18	69.79	85.74	80.87	85.24	85.66	93.08
	Tokyobrillia tamamegaseta	76.78	75.64	70.59	69.34	86.99	79.28	85.39	83.48	88.84
	Tvetenia calvescens	78.11	76.17	72.07	68.77	87.65	81.84	86.00	85.16	90.80
	Tvetenia tamaflava	76.13	81.57	76.91	73.42	94.39	78.82	83.39	82.23	85.04
	Xiaomyia sp. 3XL	78.63	76.82	70.33	68.45	91.67	79.03	83.70	81.90	88.65
	Xylotopus amamiapiatus	79.79	77.93	72.61	69.91	91.27	84.43	86.83	84.11	96.06
	Xylotopus burmanesis	77.02	75.19	70.79	69.90	84.89	79.34	83.79	82.59	94.08
G+C%	*Acricotopus zhalingensis*	23.14	22.80	27.37	29.52	11.50	20.55	14.60	16.81	15.80
	Brillia bifida	20.45	21.37	25.51	28.25	10.35	17.11	13.29	13.59	9.28
	Brillia flavifrons	24.48	24.00	28.44	31.70	11.86	19.66	18.52	20.10	9.58
	Brillia japonica	19.58	21.80	26.83	30.22	8.35	18.75	13.25	14.90	6.14
	Brillia sp. 1XL	22.48	23.58	29.15	31.12	10.47	21.34	15.55	17.89	5.39
	Brillia sp. 2XL	20.70	21.92	27.51	30.61	7.64	17.28	14.83	16.11	10.17
	Brillia sp. 3XL	21.89	24.53	28.89	31.44	13.25	20.16	14.89	16.42	9.94
	Brillia sp. 4XL	20.76	22.03	27.09	30.11	8.89	20.32	15.43	18.30	6.09
	Bryophaenocladius mucronatus	24.22	24.60	29.44	32.07	12.28	22.47	16.26	17.76	8.29
	Camptocladius stercorarius	21.32	22.13	28.20	30.58	7.62	18.39	15.32	16.75	10.41
	Chaetocladius melaleucus	22.39	23.81	28.74	31.36	11.32	21.04	15.50	18.09	14.90
	Chaetocladius oyabevenustus	20.70	22.44	28.36	30.75	8.20	20.34	15.41	17.45	5.99
	Cladotanytarsus pseudomancus	21.99	24.98	28.69	31.46	14.80	17.34	13.82	15.75	7.59
	Compteromesa sp. 1XL	23.86	25.40	31.11	33.60	11.48	23.93	16.85	22.07	7.52
	Compterosmittia nerius	21.47	18.51	23.37	26.93	5.22	20.53	15.80	16.19	7.37
	Corynoneura arctica	21.67	23.00	28.91	30.17	9.91	20.34	14.54	16.58	6.85
	Corynoneura isigaheius	20.98	22.20	27.58	30.25	8.78	19.49	16.59	18.47	8.57
	Corynoneura latusatra	19.89	22.09	27.11	29.59	9.58	18.4	13.15	14.58	9.11
	Diplocladius cultriger	22.38	23.85	29.18	30.42	11.94	21.96	15.29	17.77	9.03
	Doithrix sp. 1XL	20.36	22.91	28.73	29.61	10.40	18.7	13.78	14.08	9.26
	Epoicocladius sp. 1XL	20.94	24.80	29.29	31.04	14.08	20.39	15.95	17.72	6.60
	Epoicocladius sp. 2XL	22.05	21.53	27.66	29.97	6.96	22.27	15.60	17.43	5.96
	Eukiefferiella cynae	20.43	25.16	28.49	31.89	15.10	20.32	15.07	17.15	5.57
	Eukiefferiella gracei	20.73	25.49	30.57	30.70	15.20	17.4	14.14	15.04	7.17
	Eukiefferiella yasunoi	24.46	26.31	30.02	32.00	16.91	21.5	15.36	17.53	20.33
	Eurycnemus cf. nozakii	22.27	21.37	26.05	30.18	7.87	19.68	14.29	16.19	10.26
	Euryhapsis fuscipropes	20.91	22.59	27.84	30.49	9.43	19.57	14.30	15.80	9.62
	Euryhapsis sp. 1XL	24.19	22.18	27.34	30.61	8.59	22.04	15.64	17.82	5.84
	Euryhapsis sp. 2XL	22.88	24.74	29.43	31.00	13.78	20.4	16.43	17.84	7.60
	Euryhapsis subviridis	21.06	22.58	27.57	30.70	9.46	17.63	14.84	15.92	13.85
	Heleniella nebulosa	15.77	23.89	28.24	31.46	11.96	14.21	9.84	12.57	5.95
	Heterotanytarsus sp. 1XL	23.05	25.00	28.18	32.00	14.80	19.74	14.98	15.57	11.19
	Heterotrissocladius marcidus	19.80	25.73	30.14	31.99	15.05	18.69	13.88	15.60	6.99
	Heterotrissocladius sp. 1XL	14.71	23.54	28.08	32.10	10.45	15.37	9.74	14.61	2.61
	Hydrobaenus dentistylus	22.65	25.69	30.40	30.41	16.28	21.2	15.93	17.29	4.46
	Krenosmittia sp. 1XL	19.87	21.57	26.09	28.81	9.80	18.52	12.03	14.60	7.26
	Limnophyes asquamatus	20.15	23.19	27.57	30.80	11.22	17.5	13.54	14.24	7.42
	Limnophyes nudus	20.06	24.24	28.26	31.69	12.77	16.93	12.97	15.56	6.37
	Manoa xianjuensis	23.85	25.85	30.43	32.60	14.51	21.43	15.43	18.32	11.59
	Mesosmittia patrihortae	20.85	23.21	27.18	30.30	12.14	17.33	14.13	17.65	6.01
	Metriocnemus picipes	20.78	21.33	26.86	30.98	6.16	19.36	14.94	15.51	7.45
	Nanocladius tamabicolor	22.04	23.81	29.29	30.89	11.26	19.72	15.00	17.69	8.18

	Neobrillia longistyla	19.88	21.81	26.03	29.21	10.17	17.53	12.31	14.17	3.33
	Parakiefferiella bathophila	24.16	26.13	29.58	31.48	17.33	18.95	15.48	17.17	15.10
	Parakiefferiella sp. 1XL	22.51	22.57	29.00	30.68	8.04	21.07	15.79	16.33	6.49
	Parakiefferiella sp. 2XL	21.40	22.54	26.79	30.03	10.81	17.63	15.24	16.24	0.00
	Parakiefferiella viktana	20.34	24.39	29.19	31.42	12.55	18.74	14.03	15.96	15.09
	Parametriocnemus scotti	23.40	24.12	28.55	31.26	12.55	18.49	16.05	15.99	27.54
	Paraphaenocladius impensus	21.70	24.22	28.99	31.47	12.21	18.89	14.87	14.96	7.24
	Pseudorthocladius cristagus	21.59	22.96	26.91	31.00	10.98	19.58	13.15	15.98	4.27
	Pseudosmittia sp. 1XL	22.55	21.94	28.42	30.42	6.98	20.81	15.73	17.29	6.88
	Rheosmittia sp. 1XL	19.87	21.86	27.02	30.19	8.38	18.85	13.63	15.94	4.67
	Rheosmittia sp. 2XL	20.76	22.86	27.95	31.18	9.44	17.94	14.18	15.69	4.61
	Shangomyia impectinata	23.43	22.35	27.89	30.60	8.55	21.41	15.87	18.64	6.27
	Smittia edwardsi	22.77	22.48	28.25	29.41	9.80	22.06	16.06	18.39	6.76
	Smittia leucopogon	20.43	24.41	28.32	30.37	14.53	20.83	15.43	17.47	7.93
	Tanytarsus verralli	22.16	23.76	26.82	30.21	14.26	19.13	14.76	14.34	6.92
	Tokyobrillia tamamegaseta	23.22	24.36	29.41	30.66	13.01	20.72	14.61	16.52	11.16
	Tvetenia calvescens	21.89	23.83	27.93	31.23	12.35	18.16	14.00	14.84	9.20
	Tvetenia tamaflava	23.87	18.43	23.09	26.58	5.61	21.18	16.61	17.77	14.96
	Xiaomyia sp. 3XL	21.37	23.18	29.67	31.55	8.33	20.97	16.30	18.10	11.35
	Xylotopus amamiapiatus	20.21	22.07	27.39	30.09	8.73	15.57	13.17	15.89	3.94
	Xylotopus burmanesis	22.98	24.81	29.21	30.10	15.11	20.66	16.21	17.41	5.92
	Acricotopus zhalingensis	0.02	-0.17	-0.06	-0.37	-0.11	0.02	-0.02	0.02	0.03
	Brillia bifida	0.01	-0.20	-0.10	-0.39	-0.12	0.05	-0.04	0.02	-0.06
	Brillia flavifrons	0.04	-0.17	-0.05	-0.40	-0.09	0.01	-0.09	-0.04	-0.03
	Brillia japonica	0.00	-0.16	-0.06	-0.39	-0.05	0.01	0.03	0.03	-0.01
	Brillia sp. 1XL	0.03	-0.17	-0.06	-0.37	-0.09	0.01	-0.04	-0.04	0.04
	Brillia sp. 2XL	0.01	-0.16	-0.04	-0.35	-0.12	0.02	-0.04	0.01	-0.02
	Brillia sp. 3XL	0.04	-0.15	-0.05	-0.39	-0.05	0.02	-0.05	-0.01	0.05
	Brillia sp. 4XL	0.02	-0.19	-0.09	-0.41	-0.10	0.02	-0.06	-0.04	0.00
	Bryophaenocladius mucronatus	0.02	-0.15	-0.06	-0.36	-0.06	0.00	0.00	-0.04	-0.03
	Camptocladius stercorarius	0.01	-0.16	-0.08	-0.36	-0.09	0.00	-0.02	0.02	0.02
	Chaetocladius melaleucus	0.00	-0.17	-0.07	-0.38	-0.08	0.01	0.01	0.03	0.07
	Chaetocladius oyabevenustus	0.00	-0.19	-0.09	-0.37	-0.14	0.02	-0.04	-0.07	-0.09
	Cladotanytarsus pseudomancus	0.01	-0.18	-0.05	-0.41	-0.11	0.05	-0.02	0.01	-0.01
	Compteromesa sp. 1XL	0.02	-0.17	-0.07	-0.38	-0.09	0.01	-0.03	-0.04	-0.01
	Compterosmittia nerius	0.02	-0.17	-0.06	-0.38	-0.09	0.02	-0.03	-0.02	-0.08
	Corynoneura arctica	0.02	-0.16	-0.07	-0.35	-0.09	0.02	-0.06	-0.02	-0.03
	Corynoneura isigaheius	0.03	-0.17	-0.05	-0.40	-0.09	0.03	-0.05	0.00	-0.01
	Corynoneura latusatra	0.00	-0.19	-0.07	-0.37	-0.14	0.03	0.00	0.07	-0.11
	Diplocladius cultriger	0.04	-0.16	-0.06	-0.36	-0.09	0.03	-0.05	-0.06	0.04
	Doithrix sp. 1XL	0.01	-0.17	-0.06	-0.37	-0.10	0.04	-0.01	0.05	-0.12
	Epoicocladius sp. 1XL	0.01	-0.16	-0.08	-0.37	-0.07	0.00	-0.07	-0.03	-0.03
	Epoicocladius sp. 2XL	0.02	-0.17	-0.06	-0.36	-0.11	0.00	-0.02	-0.07	-0.01
	Eukiefferiella cynae	0.02	-0.17	-0.05	-0.39	-0.10	0.03	-0.06	-0.05	0.03
	Eukiefferiella gracei	0.01	-0.16	-0.06	-0.39	-0.06	0.03	-0.08	0.05	0.02
	Eukiefferiella yasunoi	0.02	-0.16	-0.05	-0.37	-0.08	0.03	-0.05	-0.01	0.05
	Eurycnemus cf. nozakii	0.03	-0.19	-0.11	-0.39	-0.09	0.03	-0.10	-0.02	-0.07
	Euryhapsis fuscipropes	0.01	-0.15	-0.03	-0.38	-0.08	0.04	-0.04	-0.03	0.01
	Euryhapsis sp. 1XL	0.03	-0.16	-0.05	-0.37	-0.09	0.02	-0.01	-0.05	-0.03
	Euryhapsis sp. 2XL	0.03	-0.17	-0.06	-0.37	-0.09	0.02	-0.06	-0.03	-0.04
	Euryhapsis subviridis	0.00	-0.17	-0.03	-0.37	-0.12	0.04	-0.02	0.00	-0.09
	Heleniella nebulosa	0.01	-0.15	-0.07	-0.38	-0.05	0.04	0.02	0.04	-0.15
AT-Skew	*Heterotanytarsus* sp. 1XL	0.04	-0.16	-0.05	-0.39	-0.07	0.02	-0.04	-0.04	-0.01
	Heterotrissocladius marcidus	-0.01	-0.16	-0.04	-0.39	-0.08	0.02	0.00	0.02	-0.01
	Heterotrissocladius sp. 1XL	0.02	-0.15	-0.05	-0.37	-0.08	0.07	-0.06	0.03	0.05
	Hydrobaenus dentistylus	0.02	-0.17	-0.05	-0.37	-0.10	0.00	-0.01	-0.05	-0.09
	Krenosmittia sp. 1XL	0.02	-0.16	-0.08	-0.38	-0.07	0.03	-0.02	0.00	-0.07
	Limnophyes asquamatus	0.01	-0.16	-0.06	-0.37	-0.09	0.04	-0.02	0.09	-0.01

	Limnophyes nudus	0.00	-0.16	-0.06	-0.40	-0.06	0.03	0.01	0.01	0.03
	Manoa xianjuensis	0.02	-0.16	-0.04	-0.38	-0.09	0.01	-0.06	-0.02	0.04
	Mesosmittia patrihortae	0.00	-0.17	-0.07	-0.38	-0.10	0.02	0.02	0.00	-0.07
	Metriocnemus picipes	0.00	-0.18	-0.10	-0.38	-0.11	0.03	0.01	0.02	0.03
	Nanocladius tamabicolor	0.03	-0.18	-0.08	-0.37	-0.10	0.02	-0.05	-0.04	-0.04
	Neobrillia longistyla	0.03	-0.19	-0.08	-0.40	-0.10	0.04	-0.06	0.04	0.02
	Parakiefferiella bathophila	0.01	-0.17	-0.02	-0.40	-0.09	0.03	-0.01	0.00	0.04
	Parakiefferiella sp. 1XL	0.02	-0.16	-0.08	-0.36	-0.08	0.02	-0.04	-0.02	-0.07
	Parakiefferiella sp. 2XL	0.01	-0.16	-0.07	-0.39	-0.07	0.02	0.01	0.03	0.04
	Parakiefferiella viktana	0.06	-0.15	-0.06	-0.37	-0.06	0.04	0.00	0.00	0.10
	Parametriocnemus scotti	0.02	-0.16	-0.04	-0.36	-0.11	0.02	-0.01	-0.01	0.05
	Paraphaenocladius impensus	0.00	-0.17	-0.04	-0.38	-0.09	0.03	-0.01	0.04	-0.02
	Pseudorthocladius cristagus	0.01	-0.17	-0.09	-0.38	-0.06	0.01	-0.04	-0.02	-0.06
	Pseudosmittia sp. 1XL	0.04	-0.15	-0.09	-0.37	-0.04	0.03	-0.04	-0.03	0.00
	Rheosmittia sp. 1XL	0.00	-0.16	-0.04	-0.37	-0.11	0.02	-0.01	0.04	0.03
	Rheosmittia sp. 2XL	0.01	-0.16	-0.04	-0.39	-0.09	0.03	-0.03	0.05	-0.02
	Shangomyia impectinata	0.04	-0.16	-0.04	-0.38	-0.09	0.00	-0.04	-0.05	0.09
	Smittia edwardsi	0.04	-0.18	-0.06	-0.36	-0.12	0.02	-0.04	-0.06	0.03
	Smittia leucopogon	0.01	-0.15	-0.05	-0.38	-0.04	0.01	-0.06	0.02	-0.06
	Tanytarsus verralli	0.00	-0.16	-0.04	-0.38	-0.07	0.04	-0.02	0.00	-0.09
	Tokyobrillia tamamegaseta	0.05	-0.17	-0.07	-0.36	-0.10	0.01	-0.08	-0.03	0.04
	Tvetenia calvescens	0.01	-0.17	-0.03	-0.40	-0.10	0.01	0.02	-0.03	0.02
	Tvetenia tamaflava	0.03	-0.17	-0.06	-0.37	-0.11	0.03	-0.05	-0.03	0.06
	Xiaomyia sp. 3XL	-0.01	-0.18	-0.06	-0.39	-0.11	0.02	-0.03	0.00	0.08
	Xylotopus amamiapiatus	0.02	-0.17	-0.10	-0.40	-0.06	0.02	0.00	-0.02	0.07
	Xylotopus burmanesis	0.01	-0.18	-0.08	-0.37	-0.10	0.02	-0.04	-0.05	-0.14
	Acricotopus zhalingensis	-0.15	0.01	0.19	-0.13	-0.03	0.11	0.29	0.20	-0.28
	Brillia bifida	-0.16	-0.02	0.17	-0.16	-0.09	0.14	0.40	0.28	-0.28
	Brillia flavifrons	-0.20	-0.05	0.22	-0.19	-0.33	0.19	0.33	0.20	0.05
	Brillia japonica	-0.16	0.01	0.21	-0.14	-0.08	0.15	0.34	0.21	-0.50
	Brillia sp. 1XL	-0.17	-0.02	0.22	-0.19	-0.14	0.12	0.34	0.23	0.04
	Brillia sp. 2XL	-0.16	-0.02	0.22	-0.18	-0.24	0.15	0.31	0.21	-0.67
	Brillia sp. 3XL	-0.15	-0.04	0.19	-0.17	-0.23	0.16	0.30	0.18	-0.24
	Brillia sp. 4XL	-0.16	-0.07	0.14	-0.17	-0.33	0.15	0.34	0.24	-0.03
	Bryophaenocladius mucronatus	-0.19	-0.04	0.19	-0.19	-0.18	0.14	0.34	0.23	-0.34
	Camptocladius stercorarius	-0.15	0.00	0.25	-0.18	-0.21	0.17	0.29	0.19	-0.64
	Chaetocladius melaleucus	-0.13	-0.03	0.21	-0.17	-0.25	0.14	0.31	0.16	-0.22
	Chaetocladius oyabevenustus	-0.18	-0.01	0.22	-0.19	-0.13	0.13	0.34	0.26	-0.49
	Cladotanytarsus pseudomancus	-0.24	-0.06	0.14	-0.17	-0.24	0.19	0.39	0.30	-0.56
	Compteromesa sp. 1XL	-0.16	0.00	0.24	-0.15	-0.19	0.16	0.30	0.22	-0.26
	Compterosmittia nerius	-0.16	-0.05	0.15	-0.19	-0.23	0.15	0.32	0.26	-0.40
	Corynoneura arctica	-0.18	-0.03	0.23	-0.19	-0.30	0.13	0.30	0.24	-0.29
	Corynoneura isigaheius	-0.14	-0.01	0.19	-0.13	-0.22	0.14	0.30	0.20	-0.28
	Corynoneura latusatra	-0.18	-0.02	0.16	-0.14	-0.17	0.14	0.32	0.16	-0.67
	Diplocladius cultriger	-0.18	-0.03	0.24	-0.19	-0.29	0.10	0.31	0.24	-0.37
	Doithrix sp. 1XL	-0.18	-0.02	0.17	-0.14	-0.20	0.16	0.35	0.21	-0.46
	Epoicocladius sp. 1XL	-0.15	-0.05	0.22	-0.20	-0.26	0.15	0.34	0.23	-0.07
	Epoicocladius sp. 2XL	-0.18	0.01	0.25	-0.17	-0.20	0.13	0.32	0.26	-0.15
	Eukiefferiella cynae	-0.16	0.00	0.24	-0.16	-0.11	0.15	0.33	0.23	-0.02
	Eukiefferiella gracei	-0.18	-0.06	0.17	-0.20	-0.24	0.14	0.32	0.22	-0.10
	Eukiefferiella yasunoi	-0.13	-0.02	0.22	-0.15	-0.19	0.14	0.22	0.18	-0.29
	Eurycnemus cf. nozakii	-0.22	-0.02	0.18	-0.15	-0.22	0.11	0.29	0.26	-0.61
	Euryhapsis fuscipropes	-0.16	-0.05	0.15	-0.18	-0.23	0.13	0.30	0.21	-0.27
	Euryhapsis sp. 1XL	-0.22	-0.03	0.17	-0.16	-0.21	0.11	0.32	0.22	-0.16
	Euryhapsis sp. 2XL	-0.18	-0.02	0.23	-0.18	-0.20	0.13	0.34	0.22	-0.20
	Euryhapsis subviridis	-0.16	-0.04	0.18	-0.16	-0.31	0.16	0.30	0.20	-0.39
	Heleniella nebulosa	-0.23	0.01	0.25	-0.14	-0.15	0.09	0.41	0.36	-0.20
GC-Skew	*Heterotanytarsus* sp. 1XL	-0.17	-0.07	0.21	-0.21	-0.33	0.13	0.32	0.23	-0.50

	Heterotrissocladius marcidus	-0.10	-0.05	0.16	-0.17	-0.20	0.17	0.31	0.17	-0.32
	Heterotrissocladius sp. 1XL	-0.16	-0.04	0.22	-0.20	-0.22	0.10	0.34	0.20	-0.08
	Hydrobaenus dentistylus	-0.19	-0.07	0.17	-0.19	-0.28	0.11	0.32	0.22	-0.11
	Krenosmittia sp. 1XL	-0.19	-0.02	0.20	-0.16	-0.22	0.14	0.34	0.32	-0.20
	Limnophyes asquamatus	-0.19	-0.01	0.24	-0.17	-0.18	0.14	0.35	0.24	-0.40
	Limnophyes nudus	-0.20	-0.02	0.21	-0.17	-0.13	0.14	0.34	0.21	-0.55
	Manoa xianjuensis	-0.13	-0.01	0.21	-0.15	-0.14	0.14	0.27	0.19	-0.19
	Mesosmittia patrihortae	-0.18	-0.06	0.15	-0.17	-0.29	0.14	0.31	0.26	-0.26
	Metriocnemus picipes	-0.14	0.01	0.23	-0.14	-0.18	0.13	0.30	0.20	-0.29
	Nanocladius tamabicolor	-0.18	-0.04	0.22	-0.20	-0.29	0.14	0.34	0.24	-0.12
	Neobrillia longistyla	-0.20	-0.06	0.15	-0.18	-0.26	0.15	0.35	0.30	0.00
	Parakiefferiella bathophila	-0.25	-0.09	0.12	-0.19	-0.28	0.21	0.36	0.26	-0.16
	Parakiefferiella sp. 1XL	-0.17	0.02	0.25	-0.16	-0.12	0.13	0.34	0.26	-0.21
	Parakiefferiella sp. 2XL	-0.20	-0.04	0.16	-0.19	-0.16	0.16	0.37	0.24	/
	Parakiefferiella viktana	-0.18	-0.07	0.18	-0.19	-0.32	0.13	0.31	0.23	-0.50
	Parametriocnemus scotti	-0.15	-0.04	0.20	-0.18	-0.27	0.12	0.29	0.24	-0.04
	Paraphaenocladius impensus	-0.18	-0.02	0.20	-0.18	-0.16	0.12	0.35	0.19	-0.53
	Pseudorthocladius cristagus	-0.18	-0.02	0.22	-0.14	-0.25	0.14	0.33	0.30	-0.29
	Pseudosmittia sp. 1XL	-0.15	0.01	0.26	-0.16	-0.25	0.18	0.31	0.25	-0.39
	Rheosmittia sp. 1XL	-0.14	0.00	0.24	-0.17	-0.15	0.15	0.33	0.23	-0.55
	Rheosmittia sp. 2XL	-0.18	-0.03	0.20	-0.17	-0.23	0.15	0.30	0.21	-0.49
	Shangomyia impectinata	-0.20	-0.04	0.18	-0.18	-0.27	0.15	0.31	0.23	-0.18
	Smittia edwardsi	-0.18	-0.01	0.20	-0.17	-0.12	0.11	0.32	0.26	-0.33
	Smittia leucopogon	-0.10	-0.02	0.20	-0.15	-0.19	0.16	0.30	0.25	-0.26
	Tanytarsus verralli	-0.21	-0.06	0.19	-0.16	-0.31	0.18	0.35	0.20	-0.18
	Tokyobrillia tamamegaseta	-0.17	-0.02	0.24	-0.20	-0.19	0.15	0.31	0.26	-0.25
	Tvetenia calvescens	-0.18	-0.04	0.16	-0.16	-0.22	0.16	0.31	0.20	-0.12
	Tvetenia tamaflava	-0.15	-0.02	0.20	-0.13	-0.38	0.16	0.32	0.20	-0.27
	Xiaomyia sp. 3XL	-0.14	0.00	0.21	-0.17	-0.13	0.14	0.32	0.19	-0.32
	Xylotopus amamiapiatus	-0.17	-0.01	0.18	-0.14	-0.18	0.13	0.29	-0.16	0.09
	Xylotopus burmanesis	-0.22	-0.03	0.20	-0.19	-0.16	0.14	0.36	0.26	-0.44
